# The TCF C-clamp DNA binding domain expands the Wnt transcriptome via alternative target recognition

**DOI:** 10.1093/nar/gku1186

**Published:** 2014-11-20

**Authors:** Nate P. Hoverter, Michael D. Zeller, Miriam M. McQuade, Angela Garibaldi, Anke Busch, Elizabeth M. Selwan, Klemens J. Hertel, Pierre Baldi, Marian L. Waterman

**Affiliations:** 1 Department of Microbiology and Molecular Genetics, University of California, Irvine, Irvine, CA 92697, USA; 2 Department of Information and Computer Science, University of California, Irvine, Irvine, CA 92697, USA

## Abstract

LEF/TCFs direct the final step in Wnt/β-catenin signalling by recruiting β-catenin to genes for activation of transcription. Ancient, non-vertebrate TCFs contain two DNA binding domains, a High Mobility Group box for recognition of the Wnt Response Element (WRE; 5′-CTTTGWWS-3′) and the C-clamp domain for recognition of the GC-rich Helper motif (5′-RCCGCC-3′). Two vertebrate TCFs (TCF-1/TCF7 and TCF-4/TCF7L2) use the C-clamp as an alternatively spliced domain to regulate cell-cycle progression, but how the C-clamp influences TCF binding and activity genome-wide is not known. Here, we used a doxycycline inducible system with ChIP-seq to assess how the C-clamp influences human TCF1 binding genome-wide. Metabolic pulse-labeling of nascent RNA with 4′Thiouridine was used with RNA-seq to connect binding to the Wnt transcriptome. We find that the C-clamp enables targeting to a greater number of gene loci for stronger occupancy and transcription regulation. The C-clamp uses Helper sites concurrently with WREs for gene targeting, but it also targets TCF1 to sites that do not have readily identifiable canonical WREs. The coupled ChIP-seq/4′Thiouridine-seq analysis identified new Wnt target genes, including additional regulators of cell proliferation. Thus, C-clamp containing isoforms of TCFs are potent transcriptional regulators with an expanded transcriptome directed by C-clamp-Helper site interactions.

## INTRODUCTION

The Wnt signaling pathway is one of several vital developmental pathways conserved in all phyla of the animal kingdom (1,2). Wnt proteins are secreted morphogens that bind to their cognate transmembrane receptor, a complex of Frizzled and LRP family proteins (3). Binding leads to the release of the central messenger β-catenin from a destruction complex so it can translocate to the nucleus (4). Nuclear β-catenin then complexes with a member of the Lymphoid Enhancer Factor/T Cell Factor (LEF/TCF) family of DNA binding proteins, and in turn, recruits chromatin modifiers and components of the general transcription machinery to activate a Wnt target gene expression program (5–7). Depending on the developmental context and the gene programs that β-catenin-LEF/TCF complexes activate, cells are directed to proliferate, self-renew or differentiate toward specific cell fates. In abnormal settings, such as when mutations in Wnt pathway components cause overactive signaling, gene expression patterns of proliferation are unbalanced. For example, early development of the majority (80%) of colon cancer cases are driven by overactive Wnt/β-catenin signaling (8,9). The targets and gene programs that are misregulated in these cells are specified by the DNA binding specificities of the LEF/TCFs.

Genetic and biochemical studies have implicated the LEF/TCF family to be the primary sequence-specific transcription factors that mediate WNT target gene activation (5,10–12). For model systems, such as *Drosophila* and *Caenorhabditis elegans*, a single TCF carries this responsibility (dTCF/pangolin and POP-1, respectively). In vertebrate systems, the LEF/TCF family has expanded in both the number of family members as well as the diversity of isoforms produced from each gene. Diversification and subfunctionalization is due in part to alternative splicing and alternative promoter usage, which interestingly does not introduce newly evolved domains but instead has made ancient, existing domains, such as the N-terminus (β-catenin binding) and C-terminus (E-tail; C-clamp) an alternative choice (13,14). In mammals, the LEF/TCF family consists of four members: TCF1 (TCF7 gene), TCF4 (TCF7L2), LEF1 (LEF1) and TCF3 (TCF7L1). Family members are often co-expressed as sets of alternatively spliced isoforms and knockout studies indicate important non-redundant functions (14).

All LEF/TCF isoforms contain a highly conserved sequence-specific High Mobility Group (HMG) DNA binding domain (DBD) that binds to a eight nucleotide DNA sequence motif frequently called a Wnt Response Element (WRE; 5′-CTTTGWWS-3′), but other domains can greatly influence DNA binding. For example, alternative splicing of the C-terminus of TCF1 and TCF4 produces an isoform (named an ‘E-tail’ isoform) that is particularly potent in its ability to regulate transcription (15–17). TCF1E has been shown to be the only LEF/TCF isoform that can strongly regulate the SP5, CDX1 and LEF1 Wnt-target promoters (15), while TCF4E was shown to regulate the CDX1 promoter (17,18). Global gene expression analysis as well as proliferation and cell-cycle analysis in colon cancer cells showed that E-tail isoforms are potent regulators of cell-cycle progression through the G1 phase of the cell cycle (15,19), whereas other isoforms of LEF/TCFs are not effective. Interestingly, the predominant form of TCF1 in colon cancer is TCF1B, a form that does not contain the E-tail. This is in contrast to its expression in normal colon cells where the E-tail form is more prevalent and is expressed as a dominant negative isoform (dnTCF1E) that does not contain the N-terminal β-catenin binding domain (16).

The E-tail isoforms of TCF1 and TCF4 have been well studied because they include a second ancient DBD called the C-clamp and because they are predominant isoforms in the intestinal epithelium (16,20–22). The C-clamp is a zinc binding domain that recognizes a GC-rich sequence motif (‘Helper site’; 5′-RCCGCCR-3′) in combination with the HMG-WRE interaction *in vivo* and *in vitro* (15,23,24). Binding of the C-clamp to Helper sites is thought to be largely responsible for observations that the E-tail isoforms of LEF/TCFs are transcriptionally potent isoforms. There are features of HMG and C-clamp cooperation that are unique, including an unusual degree of flexibility in the spacing and orientation between the WRE and the Helper site; Helper sites can be located 5′ or 3′ of WREs and the spacing between motifs can vary from 1 nucleotide to 11 nucleotides (20,23). While *in vitro* studies and transient transfection experiments confirm that this degree of flexibility can be tolerated, the extent to which the C-clamp-Helper site interaction contributes to genome-wide binding and transcriptional regulation of Wnt target genes is unknown. Additionally, the pattern of co-occurring WREs and Helper sites in the context of chromatin has not been studied and therefore the constraints on the allowable distance and orientation between Helper and WRE sites for functional synergy are poorly understood. Finally, since only a few target genes have been identified where the C-clamp makes an essential contribution, a genome-wide analysis is needed to reveal not only patterns of binding, but to identify gene programs that are directly targeted by these ancient isoforms.

We performed ChIP-seq experiments to determine the binding profile of a wild-type and C-clamp mutant version of TCF1 in DLD-1 colon cancer cells. Our results indicate that the C-clamp-Helper site interaction widely contributes to binding site selection and strength. We find that wild-type TCF1 bound to a greater number of gene bodies and promoters compared to mutant TCF1, suggesting that the C-clamp helps position TCF1 for transcriptional regulation. Our bioinformatics analysis also indicates that the C-clamp forms of TCF1 can utilize Helper site(s) for transcription regulation independent of any obvious canonical WRE motif. We also assessed early changes in gene expression in tandem with ChIP-seq experiments using a metabolic labeling and high-throughput RNA-seq technique called 4′Thiouridine-seq. This technique selectively labels actively transcribed nascent RNAs, and by comparing 4′Thiouridine-seq transcript changes with ChIP-seq data, we identified new C-clamp-dependent Wnt target genes that showed direct regulation by TCF1, including histone genes, and other genes with high relevance to cell proliferation. We conclude that the C-clamp enables gene regulation independent of canonical WRE interactions in modulating the DNA activities and transcriptional output of TCF1E. However, our results also indicate that the DNA binding specificities of the HMG box and C-clamp synergize to control gene expression of a subset of Wnt target genes.

## MATERIALS AND METHODS

### Establishment of inducible DLD-1 colon cancer cells

The establishment of inducible dnTCF1EWT and dnTCF1Emut DLD-1 cell lines has been described previously (15).

### ChIP-Seq and ChIP validation

See Supplementary Methods. ChIP-seq data set was deposited to GEO with accession GSE53536.

### 4′Thiouridine-seq

See Supplementary Methods.

### Plasmids

Construction of TCF1EWT, TCF1Emut, expression plasmids was described previously (25,26).

### Luciferase assay

COS-1 cells were transiently transfected with BioT transfection reagent according to the manufacturer's protocol (Bioland Scientific LLC). COS-1 cells were plated at a density of 200 000 cells/well in 6-well plates 20 h before transfection. Luciferase reporter constructs (0.4 ug) were cotransfected with β-catenin (0.4 ug), β-galactosidase (0.1 ug) and a LEF/TCF expression vector (0.1 ug). Cells were harvested after 20 h, and luciferase and β-galactosidase activities were determined as described (25).

### Electrophoretic Mobility Shift Assay (EMSA)

EMSAs were carried out with 1 ng (∼200 cps) of radioactive oligonucleotide in a final reaction volume of 20 ul containing 10 mM HEPES (pH 8.0), 2.5 mM ethylenediaminetetraacetic acid (EDTA), 10% glycerol, 20 mM KCl, 5 mM MgCl2, 0.024 ug/ul salmon sperm DNA and 20 mM dithiothreitol (DTT). COS-1 cells were transiently transfected with EVR2 and expression vectors for full-length human TCF1EWT. COS-1 cells were prepared 48 h after transfection by swelling cells on ice, immersing them for 15 min in hypotonic lysis buffer (10 mM Tris pH 7.9, 50 mM KCl, 10 mM MgCl2, 0.01 mM EDTA, 1 mM DTT, 0.01 mM EGTA, 1 mM phenylmethylsulfonyl fluoride, protease inhibitor cocktail), and douncing. The methylated probe was ordered from Fisher Scientific and contains four methylated CpGs (two on each strand in the Helper sites).

## RESULTS

### ChIP-seq of dnTCF1EWT and dnTCF1Emut

Most ChIP-seq studies are descriptive studies that focus on establishing the binding profiles of one or several DNA binding proteins. However, ChIP-seq experiments are well suited to testing the functional contribution of individual protein domains within a DNA binding protein. For example, it was shown that the genome-wide binding profile of the E2F transcription factor in MCF-7 breast cancer cells is not mediated through protein–protein interaction domains, but rather almost exclusively through the E2F DBD (27). Interestingly, while there are a significant number of transcription regulators that have more than one DBD (28,29), to our knowledge there are no studies that examine the relative contribution of one DBD versus another in terms of genome-wide binding patterns. Furthermore, the coupling of this analysis to high-throughput detection of nascent transcripts is an emerging approach with few, if any, published examples. While this is a similar approach to integrating ChIP-seq with microarray or RNA-seq data sets, this technique allows for more sensitive detection of newly transcribed RNA (30).

**Figure 1.**
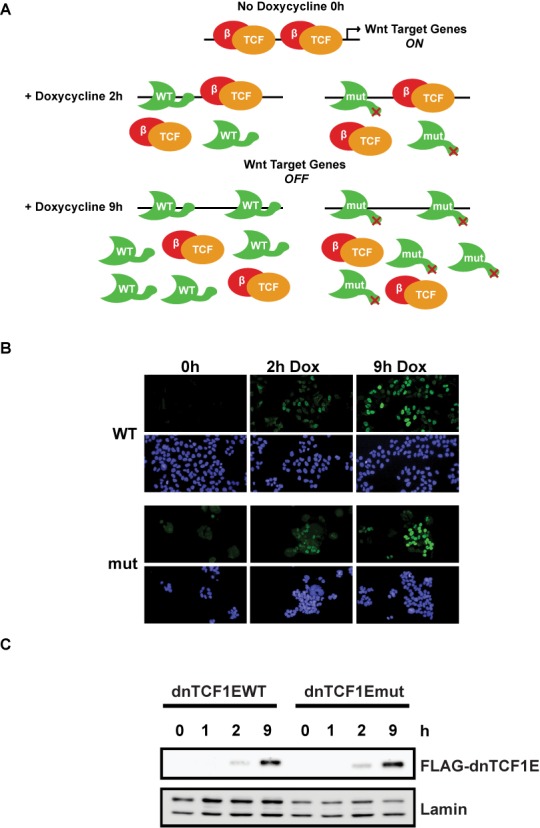
dnTCF1EWT and dnTCF1Emut doxycycline inducible system in DLD-1 colon cancer cells. (A) In the absence of doxycycline, dnTCF1E is not produced and high levels of β-catenin (red) signaling activate Wnt target gene expression. At 2 h of doxycycline induction, low amounts of dnTCF1EWT and dnTCF1Emut (green) are produced, which compete with endogenous LEF/TCF factors (orange) for binding sites and cause a downregulation of Wnt target gene expression. At 9 h post-induction, there are higher levels of dnTCF1E induced, which cause increased repression of Wnt target gene expression. (B) Immunofluorescence images of dnTCF1EWT and dnTCF1Emut expression in DLD-1 cells. dnTCF1EWT and dnTCF1Emut (green) are found exclusively in the nucleus (stained blue with DAPI). (C) Western blot analysis of a Doxycycline induction time course of FLAG-dnTCF1EWT and FLAG-dnTCF1Emut expression.

To determine the contribution of the C-clamp domain to the genome-wide binding pattern of TCF1 binding, ChIP-seq experiments were performed with a well established, doxycycline-inducible system in DLD-1 colon cancer cells (15,19,31). DLD-1 cells have high levels of endogenous Wnt signaling and therefore constitutive occupancy of Wnt target sites by endogenous β-catenin-LEF/TCF complexes. The doxycycline inducible system takes advantage of a dominant negative form of TCF1E (dnTCF1E) which when expressed in colon cancer cells, can interfere with endogenous β-catenin-LEF/TCF complexes by competing for occupancy of binding sites throughout the genome (Figure 1A) (31). Competition results in a downregulation of Wnt target gene expression because β-catenin and its transcriptional co-activators are displaced from regulatory sites (15,19) (Figure 1A). This system was first established by van de Wetering *et al.* to discover that either dnTCF1E or dnTCF4E expression causes a stall in the G1 phase of the cell cycle (19). We subsequently used this system to show that the stall requires the C-clamp domain in the E-tail (15). A microarray analysis of gene expression in parallel cell lines with either induced wild-type dnTCF1E (dnTCF1EWT) or a mutant version which has a five amino acid substitution in the C-clamp rendering it null for DNA binding (dnTCF1Emut), identified C-clamp-dependent changes in gene expression, changes that were connected to cell-cycle progression and proliferation (15). dnTCF1Emut is equally capable as dnTCF1EWT of regulating WRE-driven Wnt reporter constructs, such as TOPTK ((15), unpublished observation), so to what extent these changes reflect differences in dnTCF1E occupancy of target genes or secondary and tertiary effects not related to binding is not fully known (20). Also, whether the C-clamp defines new patterns of sequence specificity and a new subclass of target gene, or whether its function is strictly auxiliary to classic WRE-dependent gene targeting is not known. We therefore used ChIP-seq comparisons of parallel doxycycline induction of dnTCF1EWT and dnTCF1Emut to assess the role of the C-clamp in the genome-wide binding patterns of TCF1.

**Figure 2.**
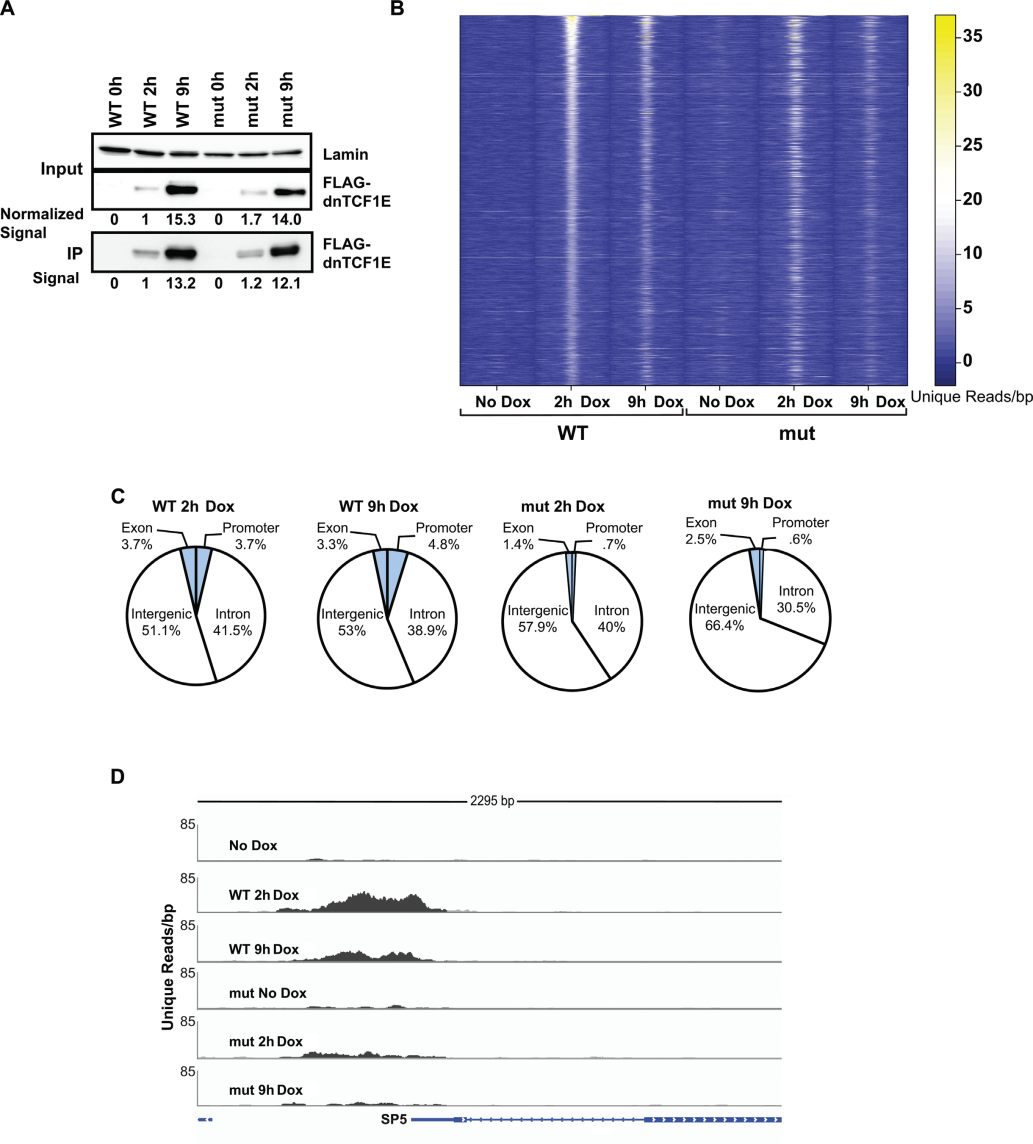
dnTCF1EWT binds more strongly to DNA and is enriched near gene loci. (A) Western blot analysis of Doxycycline induction and immunoprecipitation of FLAG-dnTCF1EWT and FLAG-dnTCF1Emut using Flag-antibody-conjugated magnetic beads. Similar levels of dnTCF1EWT and dnTCF1Emut were immunoprecipitated as quantitated by digital image analysis. Induction levels of cellular FLAG-tagged protein were normalized to Lamin and values are indicated below the panel. Immunoprecipitated levels of FLAG-tagged protein derive from the digital signal intensity of bands in the bottom panel. (B) The top scoring 1000 peaks from the dnTCF1EWT 2 h analysis were sorted by read intensity and displayed in a heat map. Read intensities (number of reads per 10 base pairs) for these regions were also displayed for the other ChIP-seq samples. The window length for each sample is 2000 bp, centered on the peak of dnTCF1E occupancy. dnTCF1E peaks were stronger at 2 h induction than 9 h induction. dnTCF1Emut was enriched at most, but not all regions that dnTCF1EWT bound. However, dnTCF1Emut enrichment at these regions was mostly weaker. (C) Distribution of the top 1000 dnTCF1EWT and dnTCF1Emut peaks. dnTCF1EWT bound to a greater number of promoter (defined as 4 kb centered over the transcription start site) and exonic regions than dnTCF1Emut. dnTCF1EWT also bound to a greater number of untranslated regions, exons and transcription termination sites, which together encompass the ‘Other’ category. (D) Example of a ChIP-seq peak at the promoter of the Wnt target gene SP5. dnTCF1EWT showed stronger binding at the SP5 promoter, consistent with its known status as a C-clamp-preferred target gene.

dnTCF1EWT and dnTCF1Emut (C-clamp mutant) were induced with doxycycline in duplicate cultures for 1 h (undetectable expression), 2 h (low amount of expression) or 9 h (higher amount of expression) (Figures 1B, C and 2A) and protein-DNA interactions were stabilized by crosslinking with formaldehyde. For each cell line, an untreated control was also included in the analysis (no doxycycline, 0 h). The early 2 h time point was included in the ChIP-seq analysis because little is known about the genome-wide binding patterns of newly translated TCFs or induced DNA binding proteins in general. In addition, the levels of immunoprecipitated wild-type and mutant protein were very similar at 2 h (Figure 2A). Much of the subsequent analysis in this study derives from this early time point. Immunoprecipitations were performed with FLAG-antibody-conjugated magnetic beads because dnTCF1EWT and dnTCF1Emut both have an N-terminal FLAG tag (a tag which we have previously shown is neutral for dnTCF1E activities (15)). Western blot analysis demonstrated that no detectable dnTCF1E was pulled down in untreated (0 h) cells and that similar levels of dnTCF1EWT and dnTCF1Emut were pulled down after 2 and 9 h of doxycycline treatment (Figure 2A). ChIP-seq was then performed in duplicate for each condition to identify regions of occupancy (see Materials and Methods). A comparison of the peaks called at each time point exhibited significant overlap for each of the dnTCF1EWT and dnTCF1Emut biological replicates. For example, the reciprocal overlap of the top 20% of dnTCF1EWT 2 h peaks was 61% and 54% and for dnTCF1Emut 2 h it was 80% and 86%. We further assessed reproducibility using scatter plot and Irreproducible Discovery Rate analysis (IDR; (32,33)). IDR analysis is a stringent rank-order approach that compares the rank order *P*-values of peaks between biological replicates and assigns a value to the concordance (or discordance) much like a false discovery rate value. IDR analysis showed that the top 1000 peaks from 2 h of Doxycline induction had a reproducibility index of 0.1 or better for the dnTCF1EWT replicates and 0.04 for the dnTCF1Emut replicates (Supplementary Figure S1D). Following the ENCODE protocol for ChIP-seq (32), we therefore pooled the biological replicates for each time point (see Supplementary Figure S1 and Supplementary Methods), and for much of the analysis that follows, only the top 1000 peaks from each of the 2 h time points was used.

A heat map representation of the distribution of read counts for the top 1000 dnTCF1EWT peaks at 2 h (out of 5580 total peaks) across all samples from this pooled data set shows sharp peaks of occupancy (Figure 2B) and known high affinity binding sites close to the peak centers (Supplementary Figure S2). The fainter heatmap signals for dnTCF1Emut at these sites indicate weaker binding, a general pattern also reflected in the fewer number of total peaks called for dnTCF1Emut (1863 total peaks) and the overall lower scores for those peaks (Supplementary Figure S2A and B). dnTCF1EWT bound to the same regions at 9 h post-induction as 2 h post-induction, however, binding was weaker at 9 h, despite greater amounts of dnTCF1E in the nucleus and immunoprecipitate at the later time point (Figures 1B and C and 2A and B). dnTCF1Emut bound to most of the same regions as dnTCF1EWT at 2 h post-induction, but binding to these regions was generally weaker, as indicated by fewer total unique reads within 500 bp of the peak centers (269.0 versus 314.2; *P* = 4.5E-16; paired *t*-test). Binding by dnTCF1Emut was also weaker at 9 h post-induction compared to 2 h post-induction, suggesting that the decrease in binding seen at later time points (even with greater amounts of dnTCF1E in the nucleus) is due to a C-clamp-independent phenomenon. To test whether dnTCF1E was inducing a repressive chromatin state that reduces binding at 9 h post-induction, ChIP-quantitative polymerase chain reaction (qPCR) experiments were performed on H3K9acetyl and H3K9me3 chromatin marks (Supplementary Figure S3A and B). H3K9me3 is associated with a closed chromatin state and decreased access of transcription factor binding (34), while H3K9acetyl is associated with an open chromatin state (35). Induction of dnTCF1EWT did not cause an increase in H3K9me3 (Supplementary Figure S3A) and unexpectedly caused an increase in H3K9acetyl by 9 h (Supplementary Figure S3B). Therefore, it is unlikely that dnTCF1E is reducing its own binding at 9 h post-induction through a chromatin-mediated mechanism. Taken together, dnTCF1EWT shows a global pattern of rapid, strong and focused binding to specific sites.

### The C-clamp binds Helper sites on a genome-wide scale and targets dnTCF1EWT to gene loci

Sites of dnTCF1EWT and dnTCF1Emut occupancy were chiefly within intergenic and intronic regions, a pattern of binding that has been observed for TCF4, a LEF/TCF family member that is co-expressed with TCF1 in colon epithelial cells (Figure 2C) (36). However, dnTCF1EWT bound more than a 5-fold greater number of promoter regions than dnTCF1mut (promoter defined as a 4 kb region centered on the transcription start site (TSS)), and 2-fold greater number of exons, indicating that the C-clamp is important for targeting dnTCF1E to regulatory sites near gene bodies. An example of a promoter-bound region is shown in Figure 2D. We have previously shown that the promoter of the Wnt target gene SP5 is strongly regulated by the C-clamp in a Helper site-dependent manner and therefore as expected, dnTCF1EWT showed strong binding to the SP5 promoter, whereas dnTCF1Emut showed weak, barely significant binding to the promoter (Figure 2D) (15).

**Figure 3.**
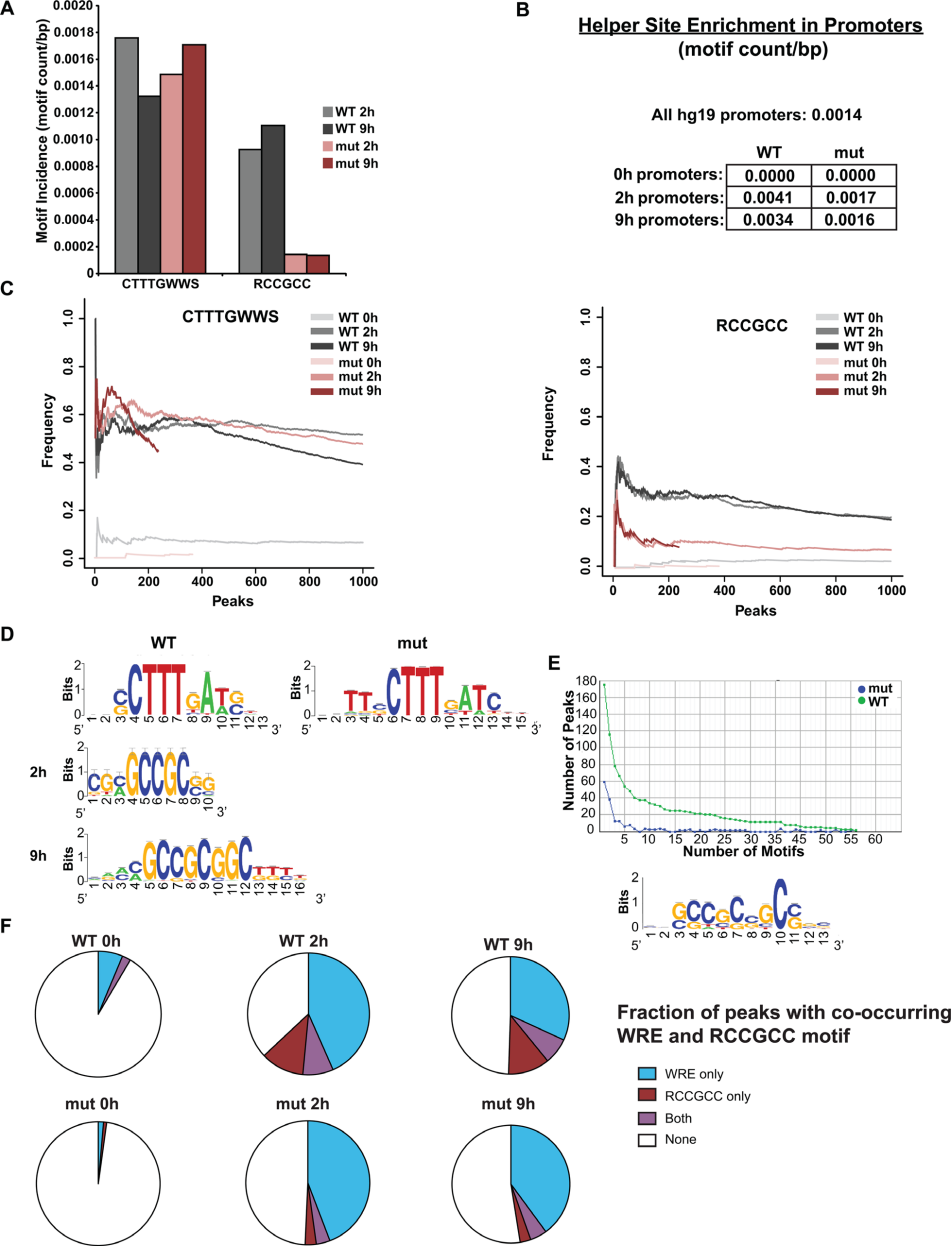
The C-clamp binds to Helper sites on a genome-wide scale. (A) The average motif incidence of the WRE (5′-CTTTGWWS-3′) and the Helper (5′-RCCGCC-3′) in the top 1000 ChIP-seq peaks was calculated by counting the incidence of the motif and dividing by the length of the peak. dnTCF1EWT and dnTCF1Emut had similar enrichment of the WRE. However, dnTCF1EWT had significant enrichment of the Helper site compared to dnTCF1Emut at 2 h (Wilcoxon–Mann–Whitney, *P* = 8.6E-23) and 9 h (*P* = 1.9E-10). (B) Helper site enrichment in promoters. The Helper site is found on average 1.4 times per 1000 bp in all human promoters (-1000 bp upstream to 100 bp downstream the TSS). Promoter regions bound by dnTCF1EWT had an enrichment of the Helper site over all human promoters (*P* = 2.3E-12), whereas dnTCF1Emut-bound promoter regions did not (*P* = 0.053). (C) Motif frequency of the WRE (left) and the Helper site (right) in dnTCF1EWT and dnTCF1Emut peaks. dnTCF1EWT and dnTCF1Emut peaks had a similar frequency of the WRE, but the Helper site was found in a greater frequency of dnTCF1EWT peaks. (D) RSAT *de novo* motif enrichment performed on dnTCF1EWT and dnTCF1Emut peaks. The WRE was the top motif found in both dnTCF1EWT and dnTCF1Emut peaks. The Helper site was the fifth most enriched motif at 2 h post-induction and the second most enriched motif at 9 h post-induction. (E) Top graph, the GC-rich motif is enriched in both frequency and copy number of dnTCF1EWT peaks compared to dnTCF1Emut peaks. A full display of *de novo* motif enrichment results is found in Supplementary Figures S4–S6. Bottom panel, the most enriched motif from RSAT differential *de novo* motif analysis which uses dnTCF1Emut ChIP-seq peaks as a background to find motifs that are comparably enriched in dnTCF1EWT peaks. (F) Fraction of the top 1000 ChIP-seq peaks with the indicated motifs.

Although the C-clamp has been shown to interact with select GC-rich Helper sites in mammals (15) and *Drosophila* (23), its role in the genome-wide binding of C-clamp isoforms of TCFs is unknown. We have previously shown that the human C-clamp interacts with a short Helper site (5′-RCCG-3′) with an unusual degree of flexibility in that the site can be recognized on the 5′ or 3′ side of a WRE with a tolerance for varied spacing between the elements (15). We also determined that the C-clamp recognizes a 7 nucleotide extended Helper site (5′-GCCGCCR-3′), a motif first identified in *Drosophila* as occurring adjacent to WREs for dTCF/pangolin recognition (23). We therefore searched for enrichment of a slightly shorter version of the extended Helper site (5′-RCCGCC-3′) in our ChIP-seq peaks because the C-clamp is highly conserved between humans and *Drosophila* and because the short 4 nucleotide Helper site occurs too frequently for meaningful searches. The ChIP-seq peaks occupied by dnTCF1EWT showed a greater total number of extended Helper sites compared to dnTCF1Emut, confirming that the C-clamp interacts with Helper sites on a genome-wide scale (Figure 3A). In contrast, dnTCF1EWT and dnTCF1Emut showed similar enrichment of the WRE in ChIP-seq peaks (Figure 3A). This suggests that the C-clamp does not interfere with the ability of the HMG DBD to bind to the WRE. To account for the issue that promoters and gene bodies tend to be more GC-rich than intergenic regions, we evaluated the occurrence of the Helper site in peaks within promoter regions (defined as -1 kb to +100 bp from the TSS) for both wild type and mutant as compared with the frequency of the Helper site in all human RNA polymerase II promoters. We used the incidence of (5′-RCCGCC-3′) per base pair to determine if the Helper site occurrence is significantly higher than expected. Despite a higher background GC content within promoters, the incidence of RCCGCC per nucleotide is still significantly greater within promoters bound by dnTCF1EWT (Figure 3B; Mann–Whitney–Wilcoxon test; *P* = 2.3E-12). For comparison, the incidence of the Helper site in promoters occupied by dnTCF1Emut is not significantly greater than the genome background promoters (*P <* 0.05; Figure 3B). We also observed a greater percentage of dnTCF1EWT ChIP-seq peaks that have at least one occurrence of the Helper site compared to dnTCF1Emut, whereas the percentage of peaks containing a WRE was similar between the two (Figure 3C). Thus, ChIP-seq analysis reveals a genome-wide association between the C-clamp and the Helper site.

For an unbiased analysis we used multiple *de novo* motif enrichment approaches including Regulatory Sequences Analysis Tools (RSAT) (37) and Hypergeometric Optimization of Motif Enrichment (HOMER) (38). These analyses demonstrated that the WRE was the most consistently enriched motif in both dnTCF1EWT and dnTCF1Emut peaks at 2 h, a result consistent with the HMG box being the dominant sequence specific DBD in dnTCF1E (Figure 3D). For the 9 h time point, the WRE continued to be highly enriched (Supplementary Figure S4), but HOMER also detected additional enriched motifs including MEF-2C, LRX, SREBP-1, -2, ATF-1 and CRX (Supplementary Figure S5A). Importantly, the Helper site was enriched in dnTCF1EWT peaks at 2 and 9 h post-induction (Figure 3D, Supplementary Figure S4). In contrast, there was no enrichment of Helper-like motifs in dnTCF1Emut peaks (Figure 3D, Supplementary Figures S4–S6) or in the 0 h controls (data not shown). To directly compare the differences between dnTCF1EWT and dnTCF1Emut we used differential *de novo* motif analysis, an approach that searches for motifs enriched in one ChIP-seq data set compared to a second data set. We determined that all motifs preferentially enriched in dnTCF1EWT versus dnTCF1Emut ChIP-seq peaks were GC-rich Helper-containing motifs (Figure 3E, Supplementary Figure S6). Interestingly, peaks with Helper sites very often did not have an identifiable WRE motif (5′-CTTTGWWS-3′; Figure 3F), suggesting that the C-clamp-Helper site interaction frequently occurs independent of canonical WRE recognition. Collectively, these different motif analyses suggest that the primary difference between dnTCF1EWT and dnTCF1Emut genome-wide binding site selection is that dnTCF1EWT utilized the C-clamp to bind regions with an enrichment of the Helper site.

### Co-occurrence of the WRE and Helper site

Previous work has established that the C-clamp binds to Helper-sites as an auxiliary domain reliant on a HMG box-WRE interaction (15,23). We therefore determined if the Helper site was enriched in dnTCF1EWT peaks that also contained a WRE (CTTTGWWS; Table 1). Both dnTCF1EWT and dnTCF1Emut contained an enrichment of peaks with 1 Helper site and 1 WRE over what was expected by chance alone (approximated using a binomial distribution, see Materials and Methods), indicating co-evolution of these two motifs. However, out of the top 1000 peaks, dnTCF1EWT had 27 peaks with 2 Helper sites and 1 WRE (*P* = 1.6E-11), while dnTCF1Emut had only 4 peaks with 2 Helper sites and 1 WRE (*P* = 0.021). dnTCF1EWT had 18 peaks with 3 or more Helper sites and 1 WRE, whereas dnTCF1Emut had 0 peaks with 3 or more Helper sites and 1 WRE. This suggests that the C-clamp can utilize multiple Helper sites to contribute to binding as has been previously suggested (20). It has also been previously suggested that despite the flexibility in orientation and spacing for the C-clamp-Helper interaction, there is a preferred orientation relative to the WRE and a selection for close proximity to the WRE (usually within 10 bp). However, an analysis of the distance between Helper sites and WREs in dnTCF1EWT ChIP-seq peaks revealed no clear pattern of orientation and distance constraints of the Helper site relative to the WRE (Supplementary Figure S7A). This again suggests that the C-clamp-Helper site interaction may occur independently of a concurrent HMG box-WRE interaction. The role of the Helper site may be to serve as a sink to bring dnTCF1EWT in closer proximity to the more transcriptionally potent WRE.

**Table 1.** Observed and expected number of the top 1000 peaks that contain multiple number of Helper sites and at least one WRE

	Number of peaks with 1+ WRE	Helper sites per peak (*N*)	Number of peaks with N+ Helper sites	Expected number of peaks with both^a^	Observed number of peaks with both	*P*-value
**WT**	506	1	193	33	80	2.6E-12
**WT**	506	2	185	4	27	1.6E-11
**WT**	506	3	37	0	11	3.3E-07
**WT**	506	4	27	0	4	0.021
**WT**	506	5	21	0	3	0.081
**mut**	423	1	54	1	32	6.6E-29
**mut**	423	2	7	0	4	0.020
**mut**	423	3	1	0	0	-
**mut**	423	4	1	0	0	-
**mut**	423	5	0	0	0	-

^a^See Materials and Methods.

### The C-clamp-Helper site contributes to ChIP-seq peak strength

**Figure 4.**
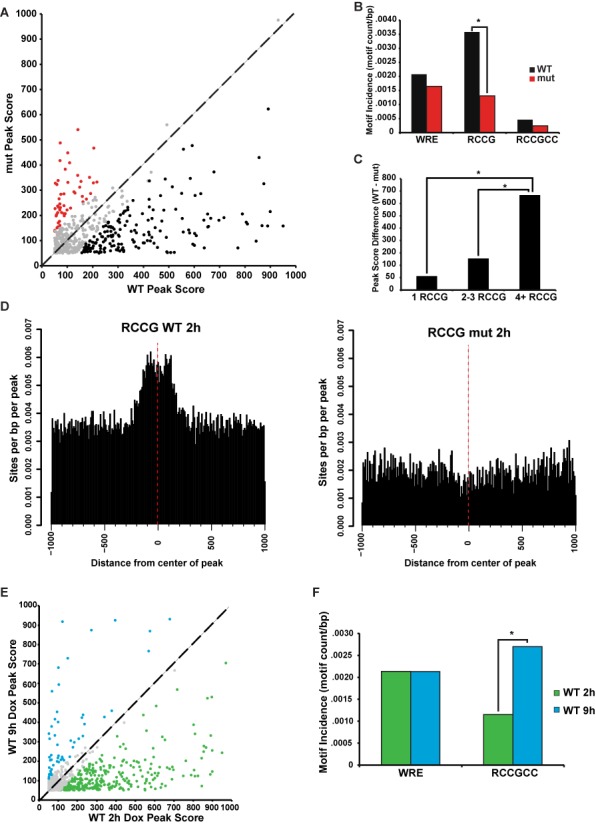
The C-clamp-Helper site interaction contributes to overall DNA binding strength. (A) Scatter plot displaying the peak scores associated with genomic regions bound by both dnTCF1EWT and dnTCF1Emut at 2 h post-induction (any overlap). dnTCF1EWT peak scores were significantly higher than dnTCF1Emut peak scores (Wilcoxon–Mann–Whitney test, *P* = 3.5E-10). Regions bound more strongly by dnTCF1EWT (black), dnTCF1Emut (red) and similarly enriched (gray) are indicated. (B) The short Helper site (5′-RCCG-3′) is significantly enriched in peaks bound more strongly by dnTCF1EWT (Wilcoxon–Mann–Whitney test, *P* = 1.3E-5), whereas the WRE is not significantly enriched in regions bound more strongly by dnTCF1EWT and dnTCF1Emut (*P* = .10). There were very few Helper sites (5′-RCCGCC’-3) bound by dnTCF1Emut and therefore a significant difference between peaks more strongly bound by wild-type and mutant was not seen (*P* = 0.97). (C) Increased copy number of the short Helper site is associated with greater differences in peak scores between dnTCF1EWT and dnTCF1Emut (1 RCCG compared to 4+ RCCG: *P* = 1.0E-7, 2–3 RCCG compared to 4+RCCG: *P* = 8.0E-6). (D) Histogram showing that the short Helper site is enriched near the center of dnTCF1EWT but not dnTCF1Emut peaks. (E) Scatter plot displaying peak scores associated with genomic regions bound by dnTCF1EWT at both 2 and 9 h of induction. Peak scores were significantly higher for dnTCF1EWT at 2 h post-induction (Wilcoxon–Mann–Whitney, *P* = 1.7E-15). (F) Regions bound more strongly by dnTCF1EWT at 9 h have an enrichment of the Helper site (*P* = 0.0008), but not the WRE (Wilcoxon–Mann–Whitney test, *P* = 0.97).

We detected 510 genomic regions that were bound both by dnTCF1EWT and dnTCF1Emut at 2 h post-induction. However, even though these regions were occupied by wild-type and mutant protein, dnTCF1EWT generally bound more strongly than dnTCF1Emut (strength defined as enrichment or peak score from MACS; *P* = 2.6E-10; Figures 2B and 4A). This difference in strength is not due to different protein levels as induction and immunoprecipitation of dnTCF1Emut was equivalent to dnTCF1EWT (Figures 1C and 2A). Motif analysis of the set of peaks bound more strongly by dnTCF1EWT revealed that the short Helper site 5′-RCCG-3′ was significantly enriched (Figure 4B). This enrichment suggests that the C-clamp-Helper site interaction contributes to dnTCF1EWT peak strength, even in regions that are bound by dnTCF1Emut and therefore presumably have strong HMG-WRE interactions. The difference in peak strengths between wild-type and mutant dnTCF1E was even more pronounced when multiple copies of the short Helper site are present (Figure 4C), suggesting that Helper site copy number makes a contribution to peak strength. The short Helper site was found near the center of wild-type but not mutant peaks (Figure 4D), consistent with the C-clamp being able to make contacts with this short motif. As previously discussed, dnTCF1EWT is generally bound to genomic regions more strongly at 2 h than it is at 9 h post-induction (Figures 2B and 4A). Interestingly, the peaks that went against this trend, that is, regions that continued to be strongly occupied by dnTCF1EWT at 9 h were peaks that had significant enrichment of the extended Helper site (Figure 4F). dnTCF1Emut also showed stronger binding at 2 h versus 9 h, but unlike wild-type protein, very few peaks remained strongly occupied at 9 h and these regions were not enriched for Helper sites (Supplementary Figure S7B). These results are consistent with the C-clamp being a DBD of moderate strength (kd = 18 nM) (20), whereas the HMG box is a stronger DBD (kd = 1 nM). At 2 h post-induction, low levels of dnTCF1EWT favor use of the HMG-WRE interaction, although the C-clamp Helper site interaction still makes an important contribution to binding (Figures 2B, 3 and 4). However, at 9 h, greater amounts of dnTCF1EWT in the nucleus allow the C-clamp-Helper site interaction to contribute more to peak strength. Taken together these analyses clearly show that the Helper site contribution to ChIP-seq peak strength is C-clamp specific.

### 4′Thiouridine-seq identifies responsive target genes of dnTCF1EWT

**Figure 5.**
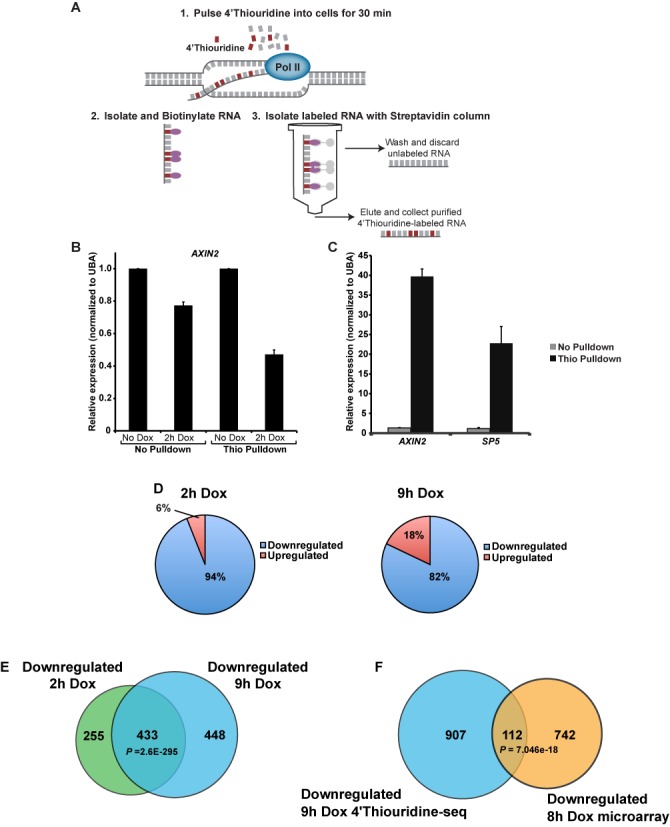
4′Thiouridine-seq identifies genes repressed by dnTCF1EWT. (A) 4′Thiouridine-seq involves addition of 4′Thiouridine into cells for 30 min to label nascent RNA transcripts. 4′Thiouridine-labeled RNA is isolated and sequenced, allowing for snapshots of transcription at any given time point of dnTCF1EWT induction. (B) Comparison of AXIN2 levels as assessed by normal RT-PCR (before pulldown) versus 4′Thiouridine-RT-PCR (same sample after pulldown), *n* = 3. Pulldown of 4′Thiouridine-incorporated transcripts causes a more dramatic decrease in AXIN2 in response to dnTCF1E WT induction to be detected (*n* = 3). (C) Wnt target genes are greatly enriched after 4′Thiouridine pulldown when compared to the housekeeping gene UBA as assessed by RT-PCR. (D) The majority of genes that changed expression after 2 h of dnTCF1EWT induction (*P* < 0.02) and 9 h of dnTCF1EWT induction (*P* < 0.012) were downregulated. (E) The overlap of genes downregulated at 2 h post-induction and 9 h post-induction was highly significant (*P*-value denotes the result of a hypergeometric test). (F) The overlap of genes downregulated at 9 h post-induction in the 4′Thiouridine-seq and at 8 h post-induction in a previous microarray experiment in a different clonal DLD-1 cell line was highly significant (*P*-value denotes the result of a hypergeometric test).

To assess the consequences of dnTCF1EWT binding on changes in gene expression, and to identify the most likely direct, regulated target genes of TCF1E, we used a metabolic labeling and high-throughput RNA sequencing technique called 4′Thiouridine-seq. 4′Thiouridine is a nucleic acid precursor that can be incorporated into nascent transcribed RNA without impairing transcription or translation (30,39). To perform 4′Thiouridine-seq, dnTCF1EWT protein was induced for 2 or 9 h and cells were pulsed with 4′Thiouridine for 30 min, allowing incorporation of the nucleotide analogue into actively transcribed nascent RNA species (Figure 5A). The 30-min labeling time was started at the same time that ChIP-seq samples were formaldehyde cross-linked so that the effects of binding on changes in gene expression could be directly correlated. Like with ChIP-seq, 4′Thiouridine-seq was performed in duplicate, and a control was included (no doxycycline). Labeled RNA was selectively purified from cell lysates, allowing for a snapshot of transcription to be taken at the 2-h time point. A distinct advantage of 4′Thiouridine-seq is the ability to detect rapid changes in transcription even if the mRNA species are stable or overwhelmingly abundant. For example, in the case of the well established Wnt target gene AXIN2, total AXIN2 mRNA levels show a modest decrease in expression after 2 h of dnTCF1E induction (Figure 5B). This is due to a lag time between changes in transcription rate and changes in total transcript levels, with RNA stability contributing to the lag time before the full magnitude of transcriptional suppression is detected. However, since the 4′Thiouridine pulldown detects nascent RNA species, it serves as a direct measure of RNA polymerase II activity during the pulse period, and in the case of AXIN2, reports a more responsive and pronounced decrease in AXIN2 expression (Figure 5B). This confirms the suitability of the 4′Thiouridine labeling and isolation scheme for RNA-seq and in particular for the matched 2 h ChIP-seq condition because stronger decreases in gene expression are more easily detected by RNA-seq. A caveat of the 4′Thiouridine-based pulldown method is that actively transcribed genes (high density of RNA polymerase and nascent RNA) will show greater overall enrichment than genes that show low transcription rates (low number of transcribing polymerases). For example, the Wnt target genes AXIN2 and SP5 are presumed to be highly transcribed in DLD-1 cells due to Wnt-activating mutations in the APC gene. Consistent with this, 4′Thiouridine labeling and pulldown caused a greater enrichment of these transcripts relative to the housekeeping gene UBA, which is not a Wnt target gene (Figure 5C). Induction of dnTCF1EWT caused a decrease in the transcription of these known Wnt target genes (AXIN2, SP5 and TNFRSF19) as assessed by 4′Thiouridine-reverse transcriptase-PCR (RT-PCR) (Supplementary Figure S8A), and transcription rates continued to decrease after 9 h of induction. Induction of dnTCF1Emut caused a similar initial decrease in transcription of AXIN2, but this was quickly followed by a recovery at 9 h post-induction (Supplementary Figure S8A). By contrast, induction of dnTCF1Emut did not significantly decrease SP5 transcription, as predicted from the weaker ChIP-seq occupancy of the SP5 promoter and our previous finding that SP5 is a C-clamp-dependent Wnt target gene (30) (Supplementary Figure S8B). Interestingly, there was still compensation in transcription rate, even an overcompensation, and as for AXIN2, this recovery was evident at the 9 h time point. These data demonstrate that the strong versus weak binding patterns of dnTCF1EWT and dnTCF1Emut connect to strong and weak effects on transcription.

### Dynamic patterns of occupancy at Wnt target genes

Purified 4′Thiouridine-labeled RNA was submitted for RNA-seq for a global analysis of effects on transcription. The great majority (94%) of the 733 genes that changed expression after 2 h of induction of dnTCF1EWT showed a decrease in transcription rate (Figure 5D), confirming that dnTCF1E is a transcriptional repressor. There was also a high degree of concordance between the two time points, as 433 genes that were downregulated at 2 h continued to be repressed at 9 h (Figure 5E), including many previously validated Wnt target genes (Table 2). Additionally, a significant overlap was observed between the 9 h downregulated genes and a previous microarray experiment at 8 h post-induction (Figure 5F) (15). Many of the ChIP-seq peaks associated with known Wnt targets were located more than 100 kb from the TSS, illustrating the difficulty in making conclusions about distal peaks and their involvement in the regulation of Wnt target gene expression. Nearly all of the known Wnt target genes that were downregulated contained at least one annotated peak from the 2 and 9 h ChIP-seq analysis (Table 2). Interestingly, a close look at Table 2 reveals that in some cases different binding sites were utilized at 2 and 9 h post-induction. For example, for AXIN2, 3 binding sites were utilized at 2 h post-induction, whereas 9 binding sites were utilized at 9 h post-induction. These 9 binding sites included the 3 that were bound at 2 h post-induction. In the case of SGK1 one binding site was located 12 239 bp from the TSS at 2 h, but at 9 h, three different sites were occupied at 16 390, 17 705 and 31 467 bp from the TSS suggesting frequent and active use of distal enhancers. This suggests that Wnt target genes contain redundant TCF binding sites and that different binding sites may be utilized depending on the chromatin conformation or the concentration of TCF.

**Table 2.** Known Wnt target genes that were downregulated and annotated ChIP-seq peaks

Wnt target gene	Fold change: 2 h, 9 h	2 h peak distance from TSS (bp)	9 h peak distance from TSS (bp)
*ASCL2*	-2.4, -3.0	-	-
*AXIN2*	-1.7, -2.8	483, 4234, 127 488	-122 214, -31 782, -4127, 550, 4401, 7366, 64 102, 127 472, 167 808
*BMP4*	-1.6, -2.6	-215 569, -152 132, -145 446	-42 955
*CDX2*	-1.9, -2.6	2396, 3221	2362, 3257
*DKK1*	-5.5, -6.7	39 631, 138 559	-
*FZD7*	-2.4, -2.6	-40 047, 34 670, 35 777	34 525, 93 752
*JAG1*	-1.4, -1.9	-32154	-45 733, -34 836
*LGR5*	N/A, -1.8	931	990, 29 670, 54 199
*MYC*	-2.4, -3.1	7022	-104 616
*PITX2*	-1.5, -1.7	-284 838, -219 399, -183 246	-422 541, -419 957, -200 616
*PPIF*	-1.4, -1.7	-	-
*SGK1*	-2.2, -2.5	12 239	16 390, 17 705, 31 467
*SOX4*	-2.4, -3.1	-208 433, -31 951, 11 965	-527 001, -174 480, -90 544, -37 651, 1316
*SOX9*	-1.6, -1.9	-160 304	746 421, -602 259, -336 893, -258 580, -199 838, -11 372
*SP5*	-2.2, -2.9	-176	-162
*TBX3*	-1.8, -1.8	-693 657, -503 686, -97 493, -84 395, 27 773, 97 991	-315 734, -283 946, -138 493, -137 130
*TCF4*	-1.3, -2.2	31 461, 134 605, 159 291	3716, 24 346, 31 485

Column titles

We assessed the overall degree in which transcription was repressed for genes downregulated at both time points. There was a significant decrease in relative transcription at 9 h versus 2 h post-induction (Supplementary Figure S9A). This same pattern was seen with Wnt target genes (Supplementary Figure S9B) and is consistent with the 4′Thio-RT-PCR results (Supplementary Figure S8). Interestingly, this trend is directly opposite from the changes in dnTCF1E peak strength (peaks were strongest at 2 h post-induction and declining by 9 h). To investigate this seemingly contradictory result, we counted the number of peaks within 50 kb of the TSS of downregulated genes for the two ChIP-seq time points (Supplementary Figure S9C). We hypothesized that decreased transcription at the 9 h time point was due to a greater number of occupied sites surrounding the gene. This was confirmed for one-third of the downregulated genes where ChIP-seq peaks were easily linked to nearby target genes. This analysis does not take into account the actions of even longer range enhancers that might be involved in the downregulation of additional genes. It is therefore possible that the stable repression of transcription derives from increased involvement of multiple regulatory sites. Additionally, there may be a lag between the initial protein binding event at 2 h post-induction and the greater changes in gene expression seen at 9 h post-induction.

**Figure 6.**
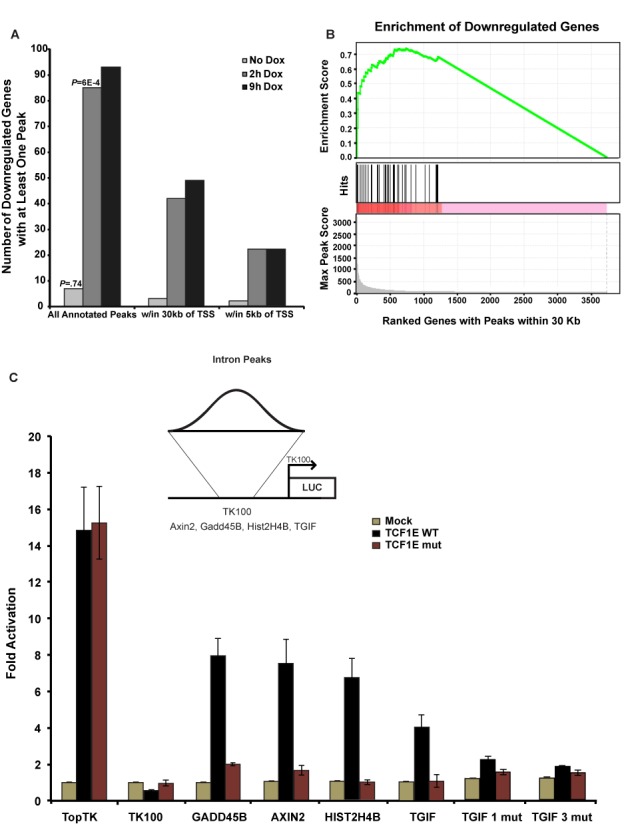
Identification of new Wnt target genes. (A) Downregulated genes with an annotated ChIP-seq peak. Note that 733 genes that had an expression change after 2 h of dnTCF1EWT induction (*P* < 0.02) were assessed for the presence of an annotated peak from the pooled analysis (peak score >67). A significant number of genes contain at least one annotated peak from the 2 h ChIP-seq time point, but not the 0 h (untreated) ChIP-seq sample. There are also many more genes that have a peak associated with the 9 h ChIP-seq than the 0 h ChIP-seq. (B) GSEA was performed on the 42 genes with at least one peak within 30 kb of the TSS as compared to the set of all genes with at least one peak within 30 kb of the TSS at 2 h post-induction and a significant enrichment (*P* < 0.05) of high scoring peaks was found. (C) Wild-type bound regions from part (A) were cloned into the Thymidine Kinase 100 (TK100) luciferase reporter. Luciferase assays were carried out in COS-1 cells with transient transfection of expression plasmids for β-catenin and full-length TCF1EWT, or TCF1Emut. Mock refers to co-transfection of an empty expression plasmid to control for β-catenin expression. Three biological replicates were included for each luciferase construct tested and luciferase/light unit values were normalized to β-galactosidase levels before comparing to normalized Mock values as described in (42). The TopTK reporter, which contains three multimerized WREs without Helper sites, was activated equally by TCF1EWT/β-catenin and TCF1Emut/β-catenin. The TK100 backbone was not activated by TCF1EWT/β-catenin or TCF1Emut/β-catenin. Helper site-containing ChIP-seq regions with one WRE (GADD45B, AXIN2) conferred activation of the reporter by TCF1EWT but not TCF1Emut. Helper site-containing regions that do not have an identifiable match to a degenerate, shorter WRE (5′-CTTTGW-3′; TGIF, HIST2H4B) also enabled activation by TCF1EWT but not TCF1Emut. Mutation of the three Helper site sequences in the TGIF region (TGIF1mut; TGIF3mut) eliminated regulation (sequences for TOPtk, GADD45B, AXIN2, HIST2H4B and TGIF and its corresponding Helper site mutations are presented in Supplementary Figure S10).

The degree to which dnTCF1EWT binding sites were linked to downregulated genes was assessed by comparing the ChIP-seq and 4′Thiouridine-seq datasets (Figure 6A). dnTCF1EWT ChIP-seq peaks were linked to 85 downregulated gene loci at 2 h post-induction (Figure 6A). In contrast, the background ChIP-seq peaks from untreated cells were associated with only seven downregulated genes. This demonstrates that dnTCF1EWT is likely involved in the direct downregulation of the majority of the 85 downregulated genes associated with a ChIP-seq peak at 2 h post-induction. Half of the peaks occupied by dnTCF-1EWT are more than 30 kb away from the TSS, suggesting that there may be widespread use of long-range chromatin loops by TCF/ß-catenin complexes (Figure 6A). Forty-two of the downregulated genes contained a ChIP-seq peak within 30 kb of their TSS (Table 3), and most of these are not currently known as Wnt target genes. We also assessed the peak distribution of peak scores of the peaks associated with the 42 downregulated genes, and performed gene set enrichment analysis (GSEA) and found a significant enrichment of strong peak scores for the peaks within 30 kb of these genes as compared to the max peak score for all genes with at least one peak within 30 kb of the TSS (Figure 6B). These represent new possible direct Wnt target genes, an illustration of the distinct advantage of combining ChIP-seq with 4′Thio-seq.

**Table 3.**
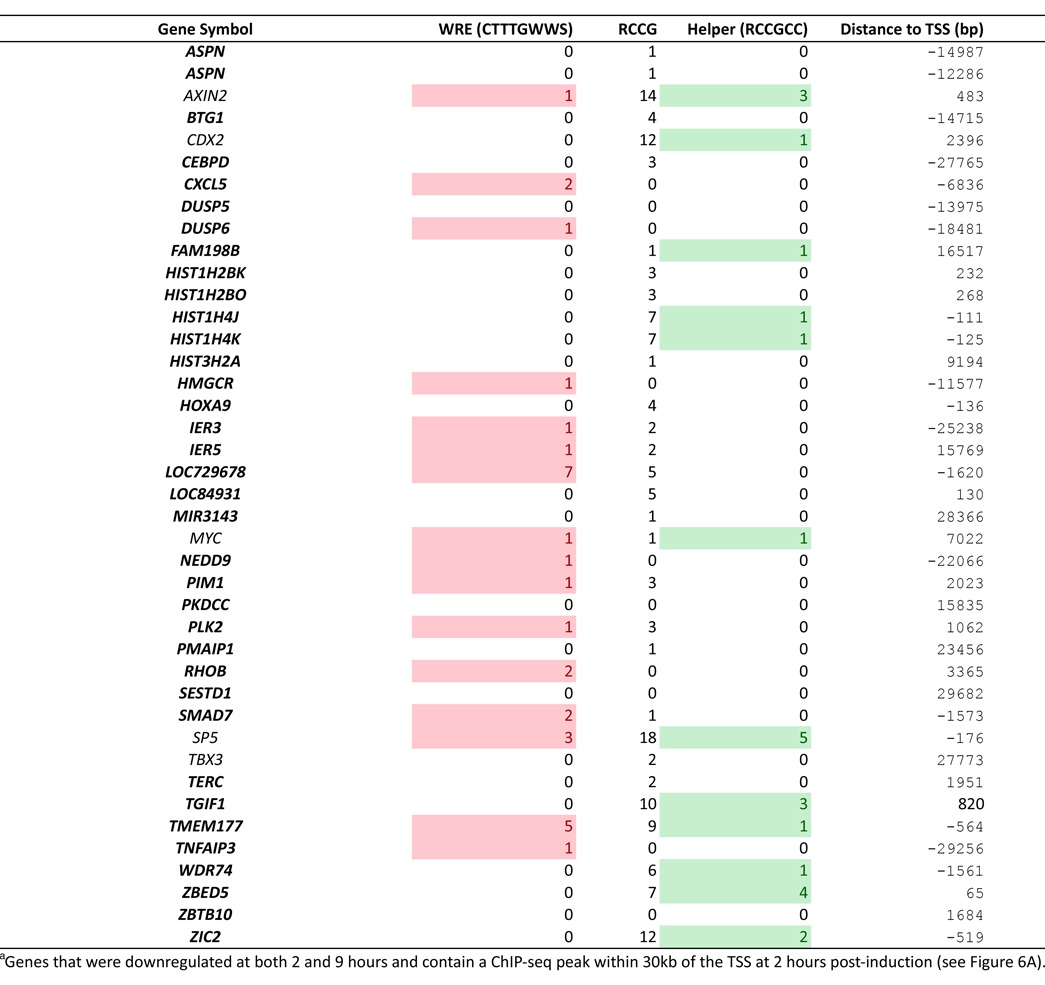
Proposed^a^ (bold) and known Wnt target genes

To test whether the ChIP-seq peaks from these potential new target genes are functional and capable of conferring β-catenin transcription regulation, we used an established transient transfection assay in COS-1 cells where transiently expressed TCF1E/β-catenin complexes specifically drive luciferase reporter gene expression when *bona fide* WREs are present in the reporter plasmid (Figure 6C; (15)). For our validation test, we subcloned nine dnTCF1EWT peaks into luciferase reporter plasmids: three regions are located within introns (GADD45B, AXIN2, TGIF), one is in an exon (HIST2H2AC), one is located at a transcription termination site (HIST2H4B), one is in an intergenic region (BAMBI) and three are within basal promoters that encompass the TSS (HIST1H4K, TMEM177, ZBED5). All nine of these peaks were specifically occupied by dnTCF1EWT, not the mutant protein, hinting that these could be new C-clamp-dependent regulatory sites. We observed that full-length TCF1EWT/β-catenin activated luciferase expression from 4 of the 9 regions (those associated with GADD45B, AXIN2, TGIF and HIST2H4B) while TCF1Emut/β-catenin could not (Figure 6C; see Supplementary Figure S10 for nucleotide sequences of the four, regulated regions). In contrast, TCF1EWT/β-catenin and TCF1Emut/β-catenin equally activated a Wnt reporter plasmid that contains multimerized copies of the WRE, but no Helper sites (see TOPTK in Figure 6C). The remaining five regions were either non-responsive, or as independent promoters, they were not active suggesting that the subcloning disrupted either the full regulatory region or chromosomal context. Similar results were recently reported where 50% of high confidence ChiP-seq peaks for β-catenin conferred Wnt regulation in a transient transfection assay (31). The positive results are also consistent with a microarray experiment that we previously reported showing that two of these four genes (GADD45B and TGIF) were regulated in a C-clamp-dependent manner (15). HIST2H4B mRNA was not detected in the microarray, likely because mature histone mRNA is not polyadenylated and is not commonly enriched for library construction and microarray analysis. Our data therefore highlights a potential new class of Wnt target gene, as the HIST2H4B region was as responsive to TCF1E/β-catenin as regions from well known, validated Wnt targets. AXIN2 is one of the well known Wnt target genes and our published microarray analysis had identified it as independent of C-clamp regulation (15). However, since there are multiple ChIP-seq peaks associated with AXIN2, it is likely that one or more of these peaks are C-clamp-dependent including the region analyzed in this study. Most surprising was the fact that two of the subcloned regions (those associated with TGIF and HIST2H4B) contain only Helper sites and no significant match to a canonical WRE motif. To exclude the possibility that WRE sites were missed by the use of a too stringent canonical motif in our sequence search (CTTTGWWS), we used the exact WRE motif developed using the WT 2 h peak data to generate a positional weight matrix with more sequence flexibility (Supplementary Figure S5C). Using this more flexible motif we repeated the search and identified a second WRE site in the AXIN2 insert. However, we found no additional matches to the more degenerate WRE in TGIF or HIST2H4B sequences. Instead, two sequences with key, important mismatches to the canonical WRE were present. Since both of these regions drove C-clamp-dependent activation of reporter gene expression, we hypothesized that the C-clamp-Helper site interactions are critical transcription regulatory motifs in regions that do not contain identifiable matches to WREs. To test this hypothesis, we introduced site-specific mutations in the three Helper sites in the TGIF peak (see Supplementary Figure S10 for the sequence and mutations). Mutation of one Helper site greatly reduced regulation β-catenin, and mutation of all three Helper sites eliminated regulation (Figure 6C).

To address whether Helper sites are sufficient to regulate transcription on a global level, we divided peaks within 30 kb of the TSS of genes detected in 4′Thiouridine-seq into several categories: peaks with no WREs or Helper sites, those with WREs, those with Helper sites and those with both Helper sites and WREs. As expected, peaks with one or more WREs were significantly associated with downregulated genes compared with peaks with no WREs (Supplementary Figure S11). In contrast, peaks with Helper sites alone were not significantly associated with downregulated genes, suggesting that WRE-independent Helper site-mediated transcriptional regulation is likely a gene-specific rather than a globalized mode of regulation. However, peaks with two or more Helper sites and a WRE were significantly associated with greater downregulation of genes compared with peaks with only WREs. Taken together, our results indicate that the C-clamp Helper site interaction synergizes with the HMG-WRE interaction for strong transcriptional control of target genes and in special cases, the C-clamp can participate in direct transcriptional regulation independent of a WRE.

## DISCUSSION

We have used ChIP-seq experiments with dnTCF1EWT and dnTCF1Emut to discover that the C-clamp-Helper site interaction plays an important role in dnTCF1E binding and target gene regulation across the genome. Several of the most notable effects of the C-clamp were its role in the targeting of dnTCF1E to promoters of polymerase II genes, the rapid and stable association of dnTCF1E with those genomic locations and the significant variation in the relative positioning of the WRE and Helper sites. The Helper site was significantly enriched in dnTCF1E peaks, but the number, orientation and the distance of those sites relative to WRE motifs were highly variable. These observations connect to *in vitro* selection experiments with oligonucleotides where the relative positions of WRE and Helper site were also variable (15). The use of 4′Thiouridine-seq confirmed that the C-clamp-Helper site interaction plays an important role in transcriptional regulation of target genes, especially when a WRE is present in the vicinity (within the same ChIP-seq peak) of the Helper site (Supplementary Figure S11). dnTCF1EWT showed stronger binding than dnTCF1Emut at most co-occupied genomic regions (Figure 4A). This occurred at many regions without an obvious Helper site, suggesting that the C-clamp strengthens the overall binding of dnTCF1EWT to DNA, or at least its interaction with chromatin. dnTCF1EWT also bound to many more promoters and gene loci than dnTCF1Emut (more than 3-fold; Figure 2C). Therefore, there seems to be at least two contributions of the C-clamp. One is to recruit dnTCF1E to CpG islands that contain copies of the Helper site and another is to increase the overall affinity of dnTCF1E for DNA.

### The C-clamp connects to Helper sites genome-wide

While the C-clamp exhibits specific DNA binding *in vitro*, our analysis is the first to test whether this specificity holds for binding to sites genome-wide in living cells. Our data demonstrate a strong correlation between the C-clamp and Helper sites. First, Helper sites were present in 30% of the top 500 strongest dnTCF1EWT peaks but only 10% of the top 500 dnTCF1Emut peaks (Figure 3C). Second, the number of Helper sites per region was much greater in dnTCF1EWT peaks than dnTCF1Emut peaks. This suggests that the C-clamp utilizes multiple copies of the Helper site for binding (Figure 3A and E), an observation consistent with other studies reporting that clusters of Helper in the vicinity of WREs was an effective criteria for *in silico* searches for Wnt target genes (22,23). Third, *de novo* motif enrichment revealed that the Helper site was specific for dnTCF1EWT peaks and not dnTCF1Emut peaks (Figure 3D and E, Supplementary Figures S4–S6). Finally, the presence of the short Helper site (5′-RCCG-3′) in regions co-occupied by dnTCF1EWT and dnTCF1Emut was associated with stronger binding by dnTCF1EWT (Figure 4B and C). Co-occurrence statistics suggest that there is significant co-enrichment of the WRE (5′-CTTTGWWS-3′) and Helper sites (5′- RCCGCC-3′) in dnTCF1EWT-bound regions, especially in those regions with multiple copies of the Helper site (Table 1). Co-occurrence of WREs and Helper sites is conserved (Supplemental Figure S7A), suggesting that the 10% of dnTCF1Emut peaks that contain both elements might derive from the co-evolution of strong WREs with Helper sites.

Motif analysis also identified an unexpected feature, which was the functional targeting of TCFs to regulatory regions without a canonical WRE. Greater than half of the dnTCF1EWT peaks with Helper sites did not contain a match to a canonical WRE, even with sequence-degenerate flexibility at the last three positions of the WRE motif (5′-CTTTGWWS-3′; Figure 3F, Supplementary File S1). This continued to be the case when the criteria was further relaxed (5′-CTTTGW-3′; Figure 6C). This was surprising given the initial reports that identified functional associations of the Helper site with the WRE in several target genes (20,22,23). A purified C-clamp protein fragment has been shown to bind independently and specifically to the Helper site *in vitro*, suggesting that the C-clamp can function as an autonomous sequence specific DBD (23,24). However, functional experiments with *Drosophila* TCF (which always contains a C-clamp) showed that a luciferase reporter plasmid with multimerized Helper sites was not responsive, whereas the Helper site greatly augmented activation of a reporter with multimerized WRE motifs (23). Nevertheless, our combined ChIP-seq/4′Thiouridine-seq analysis identified 27 candidate C-clamp-dependent genes that contain a ChIP-seq peak within 30 kb of the TSS and were downregulated selectively by dnTCF1EWT. The occupied peaks surrounding most of these genes do not contain obvious canonical WREs, whereas they have at least one or more Helper sites (Supplementary File S3). Our luciferase validation experiments demonstrated that at least two of these peaks (from the TGIF and HIST2H4B genes) conferred transcriptional regulation by full-length TCF1E/β-catenin complexes (Figure 6C). These results suggest that the C-clamp-Helper site interaction enables biologically relevant regulation of target genes in the absence of an identifiable WRE. Therefore, the difference between multimerized Helper sites and a native region of the genome in conferring C-clamp-dependent regulation may have to do with the fact that native binding sites in the context of chromatin convey additional regulatory information. For example, it is important to note that the absence of an obvious WRE does not mean that the HMG DBD is not participating in binding to other native sequences. As a class of DBD, HMG boxes are flexible in sequence recognition, showing at most a 40-fold difference between specific and non-specific binding (40) and the ability to recognize bent DNA structures. This relaxed DNA binding property implies that the HMG DBD is likely to be a constant contributor to DNA binding, just not always to recognizable WREs. Interestingly, *de novo* motif enrichment analysis of the ChIP-seq peaks shows that Helper sites are often flanked by A-T tracks, a sequence prone to bending and known to be recognized by HMG box DBDs (Supplementary Figure S6) (41). Therefore, the HMG box and the C-clamp likely can bind coordinately to DNA, but the presence of the C-clamp may enable HMG recognition of highly degenerate sequences possibly including T-tracts that do not function as independent, functional WREs on their own (15). An alternative explanation is that the C-clamp engages in protein–protein interactions for recruitment to some of the sites that do not have WREs (42). Our data do not rule out this possibility, however, given that purified C-clamp protein fragments bind specifically to the same Helper site sequence motif that we find enriched in ChIP-seq peaks, we propose that we have identified a non-WRE motif responsible for some of the direct genome-wide binding patterns of TCF. This has implications for the discovery of new Wnt target genes, which are often determined partly through searches for high scoring canonical WREs in regulatory regions.

### TCFs exhibit dynamic binding to target sites

Our analysis also identified a dynamic pattern of genome occupancy where both dnTCF1EWT and dnTCF1Emut binding decreased at 9 h post-induction (Figure 2B). Given that all transcription factors exhibit a dynamic on/off rate of binding to target sites, we hypothesized that dnTCF1E might induce a change in chromatin conformation which restricts binding at later time points. However, ChIP-qPCR analysis of repressive chromatin marks do not support this hypothesis (Supplementary Figure S3A and B). Another possible source for this dynamic shift could be a compensatory response where increased DNA binding of other LEF/TCF family members competes with the induced dnTCF1E for binding at the later time points. While this is certainly possible, western blot analysis does not show any increases in the expression of other LEF/TCF family members after induction of dnTCF1E (data not shown). Another possible source for this phenomenon could derive from the experimental design where the increased number of genomic binding sites at later time points (∼5700 sites at 2 h versus ∼13 000 sites at 9 h) means that the early binding sites are proportionally less represented in the sequencing analysis of the later 9 h time point. However, it is unclear why this result is also evident in our ChIP-qPCR validation experiments because qPCR utilizes primers to survey specific enriched regions (Supplementary Figure 3C–F). Another possible source for the decreased binding at 9 h is post-translational modification of dnTCF1E. For example, TAK/NLK proteins have been shown to phosphorylate LEF/TCFs, causing their disassociation from the DNA template (43). Therefore, the decrease in binding at later time points could be due to a stress response, where high levels of dnTCF1E cause the upregulation of a kinase or other regulatory partner that is capable of removing dnTCF1E from the DNA template.

In addition to the dynamic temporal shifts of occupancy, the DNA sequence of the Helper site suggests another way in which TCF binding can be modulated. Embedded in each DNA strand of the Helper site sequence is one CpG dinucleotide, whereas the WRE does not have any CpG pairs. It is possible that the portion of the Wnt transcriptome that depends on C-clamp activity might be rendered inaccessible through DNA methylation. A preliminary test of this notion was carried out by an EMSA of TCF-1EWT binding to an oligonucleotide probe encoding two WREs and two Helper sites (Supplementary Figure S12). A mutation in the Helper site reduced binding to the oligonucleotide, but not as much as a version of the EMSA probe that contained a methylated CpG dinucleotide in both strands of the Helper site. Binding was strongly reduced even though the consensus, canonical WRE remained the same in all the probes. Thus, targeted methylation of Helper sites in the genome might be one way in which the Wnt transcriptome can be shaped by epigenetic modification.

### Linking ChIP-seq and 4′Thio-seq identifies direct target genes

4′Thiouridine-seq was a useful technique for identifying immediate decreases in transcription in response to induction of dnTCF1EWT. Combining this data with our ChIP-seq results enabled us to link hundreds of dnTCF1EWT occupied regions to stably downregulated genes and therefore prompted predictions of additional direct target genes (Supplementary File S4, and also see Table 3). 4′Thiouridine-seq was particularly well suited for detecting the consequence of dnTCF1EWT binding at 2 h post-induction when induced protein was barely detectable (Figure 6A). Many genes were downregulated by dnTCF1EWT at both 2 and 9 h post-induction including many known Wnt target genes (Table 2). However, genes were downregulated more strongly at 9 h post-induction than 2 h (Supplementary Figure S9A and B), a surprising result given that peaks of dnTCF1EWT occupancy were generally strongest at the 2 h time point. The reason for the apparent discrepancy between occupancy and transcription is not fully understood, however, we note that the number of distinct genomic regions occupied by dnTCF1EWT that are within a distance cut-off of 50 kb relative to target genes increases at 9 h post-induction, a pattern likely due to increased concentration of dnTCF1EWT protein in the nucleus (Figure 1C and Supplementary Figure S9C). Thus, Wnt target genes might be regulated from multiple redundant binding sites with the concentration of LEF/TCFs playing a role in which sites will be utilized for regulation.

The great majority of the downregulated genes are not known Wnt target genes. This is even true after 2 h post-induction when indirect effects have not had as much time to accumulate and therefore direct gene expression changes should be the most enriched. The identification of downregulated genes that contain closely linked ChIP-seq peaks at 2 h post-induction is strong evidence that our analysis identified new Wnt target genes (see Table 3). Database for Annotation, Visualization and Integrated Discovery analysis of these genes revealed an ontology enrichment of genes that are involved in regulation of cell proliferation: cell cycle, Wnt signalling, TGFβ/BMP signaling and embryonic/morphogenesis processes (Supplementary File S5), consistent with the known role of Wnt signaling in proliferation and differentiation. We previously showed that the C-clamp controls a gene expression program that is critical to the G1-S phase transition in DLD-1 colon cancer cells through regulation of the cyclin-dependent kinase inhibitor p21 (15,19). That our analysis identified multiple histone genes hints that Wnt signaling may also target this class of gene to prepare cells for the S phase of the cell cycle when a newly replicated daughter genome must be packaged with nucleosomal histones. Our validation focused on one ChIP-seq peak associated with one histone gene. However, transcription of over 20 different histone genes was inhibited by dnTCF1EWT hinting that regulation may be more widespread, a new finding that has not been previously reported. Histone mRNA ends in a stem-loop sequence and, therefore, this class of Wnt target gene may have been largely undetected in prior transcriptome studies that enrich for polyadenylated, RNA polymerase II transcripts. Taken together our discovery highlights a distinct advantage of the 4′thiouridine-seq method, which enriches for nascently transcribed RNA regardless of polyadenylation status.

## SUPPLEMENTARY DATA

Supplementary Data are available at NAR Online.

SUPPLEMENTARY DATA
