# Supplementary material for: The TCF C-clamp DNA binding domain expands the Wnt transcriptome via alternative target recognition
Source: Nucleic Acids Res. 2014 Nov 20;42(22):13615–32. doi: 10.1093/nar/gku1186 (PMC4267635; doi:10.1093/nar/gku1186)
Supplement: SUPPLEMENTARY DATA [file supp_42_22_13615__index.html]

The TCF C-clamp DNA binding domain expands the Wnt transcriptome via alternative target recognition — The TCF C-clamp DNA binding domain expands the Wnt transcriptome via alternative target recognition — SUPPLEMENTARY DATA 

# The TCF C-clamp DNA binding domain expands the Wnt transcriptome via alternative target recognition

## SUPPLEMENTARY DATA

**Files in this Data Supplement:**

- SUPPLEMENTARY DATA
- SUPPLEMENTARY DATA
- SUPPLEMENTARY DATA
- SUPPLEMENTARY DATA
- SUPPLEMENTARY DATA
- SUPPLEMENTARY DATA
