# Supplementary material for: The TCF C-clamp DNA binding domain expands the Wnt transcriptome via alternative target recognition
Source: Nucleic Acids Res. 2014 Nov 20;42(22):13615–32. doi: 10.1093/nar/gku1186 (PMC4267635; doi:10.1093/nar/gku1186)

SUPPLEMENTARY METHODS

ChIP-seq:

dnTCF1EWT and dnTCF1Emut stable DLD-1 cells were grown in doxycycline-free RPMI complete medium. ChIP-seq experiments were performed in duplicate, and for each replicate, 9 million cells were seeded in 15cm plates (3 million cells in three 15cm plates). After 48 hours, RPMI media was supplemented with 10 ug/mL blasticidin and 300 ug/mL zeocin for 24 hours to ensure selection of cells that express the dnTCF transgene. Cells were then refreshed with RPMI complete medium without blasticidin or zeocin for another 24 hours. dnTCF1EWT and dnTCF1Emut cells were induced with 1 ug/mL doxycycline in RPMI (complete) medium without blasticidin or zeocin. Control and dnTCF1E-induced cells were collected and resuspended in 1% formaldehyde in 1X PBS for 12 minutes with gentle shaking. Crosslinking was quenched with 125mM glycine for 5 minutes. Crosslinked cells were collected by centrifugation and washed with 1X PBS to remove excess formaldehyde. Cell pellets were frozen at -80°C. Frozen pellets were thawed on ice for 10 minutes and resuspended in 3 mL LB1 buffer (50 mM Hepes-KOH pH 7.5, 140 mM NaCl, 1 mM EDTA, 10% glycerol, 0.5% NP-40, 0.25% Triton X-100, protease inhibitor cocktail) with shaking at 4°C for 5 minutes followed by centrifugation. Pellets were then resuspended in 3 mL LB2 buffer (10 mM Tris-HCl pH 8.0, 200 mM NaCl, 1 mM EDTA pH 8.0, 0.5 mM EGTA pH 8.0, protease inhibitor cocktail) and centrifuged again. Pellets were resupended in LB3 buffer (10 mM Tris-HCl pH 8.0, 200 mM NaCl, 1mM EDTA pH 8.0, 0.5 mM EGTA pH 8.0, 0.1% Na-deoxycholate, 0.5% N-lauroylsarcosine, protease inhibitor cocktail) 3 times. After 3 cycles of resuspension in LB3 and centrifugation, pellets were resuspended in 2.5mL LB3. The resulting lysates were submitted to the UC Irvine Genomics High-Throughput Facility for sonication. Sonication was performed with the Covaris AFA sonicator for 30 minutes at 4°C on the “High" setting. Sonicated samples were centrifuged and supernatants were collected. For the Input samples, 100 ul of the untreated (no doxycycline) lysates were aliquotted and 45 ul of Proteinase K buffer (30mM Tris-HCl pH 8.0), 5 ul of 20 mg/mL Proteinase K, and 9 ul of 5M NaCl were added to the mix. Crosslinking was reversed at 65°C overnight and DNA was purified with the Fermentas GeneJET PCR Purification Kit. For immunoprecipitations, Bradford assays were first performed to determine the concentration of each lysate (2ul lysate was added to 1 mL of Bradford reagent for 10 minutes). The resulting reading was used to calculate how much lysate was needed for each immunoprecipitation. Based on empirical trials, it was determined that 500 Arbitrary Units (AU) worth of lysate gave good ChIP enrichment (unadjusted Bradford reading multiplied by “X” ul of lysate = 500 AU). Typically, 1.5 mL to 2 mL of lysate was used for each immunoprecipitation. Before immunoprecipitation, lysates were precleared with 30 ul His-magnetic beads (Invitrogen 10103D) for 30 minutes. Lysates were then separated from His-magnetic beads and added to the immunoprecipitation reaction. 50 ul of FLAG-antibody-conjugated magnetic bead slurry (Sigma A2220) was used for each immunoprecipitation. Beads

were washed 3 times with 1 mL 1X PBS/BSA solution (5 mg/mL BSA Fraction V in 1X PBS) before lysates were added. Immunoprecipitations were carried out overnight at 4°C with shaking. Beads were then magnetically separated and washed 2 times with 1 mL LiCl buffer (100 mM Tris pH 7.5, 500 mM LiCl, 1% NP-40, 1% sodium deoxycholate) with shaking at 4°C for 5 minutes. Beads were then washed 2 times with cold 1X TBS and on the last wash beads were transferred to a new tube. Beads were resuspended in Proteinase K buffer (30mM Tris-HCl pH 8.0) and 0.6mg/mL Proteinase K was added directly to the beads (due to difficulties with elution of FLAG-dnTCF1E off the beads). NaCl was added to the bead mixture to a concentration of 300mM and samples were placed at 65°C overnight. The next day, ChIP DNA was recovered with the Fermentas GeneJET PCR Purification Kit. ChIP library preparations were performed at the UC Irvine High-Throughput genomic facility. Samples were multiplexed, with 8 samples per sequencing lane (e.g. two Input samples, two untreated samples, two 2 hour doxycycline samples, and two 9 hour doxycycline samples). Due to a library preparation issue, only one dnTCF1Emut Input library was sequenced (although at twice the depth as the other samples). Sequencing was performed on the Illumina HiSeq 2500 platform with 50 bp single end reads and resulted in approximately 25 million reads per sample. ChIP-seq sequencing reads were analyzed by first aligning 50bp single-end reads to the hg19 reference genome (GRCh37/hg19) with Bowtie v2.0.0-beta6 (1); 70-80% of the reads uniquely mapped to the genome), allowing at most three mismatches per read. MACS v1.4 (2) was used to call ChIP-seq peaks where only one unique read per position was retained to avoid PCR artifacts and a default cut-off for a peak score greater than 50 was used to discard weak binding events. Replicate mapped reads were either pooled together before peak calling (pooled analysis) or replicates were kept separate and peak calling was performed independently (replicate analysis). For ChIP on chromatin marks, the Abcam X-ChIP protocol (available online) was used with an anti H3K9 acetyl antibody (ab4441) and an anti H3K9me3 (trimethylation) antibody (ab8898) from Abcam.

ChIP-qPCR primers
SP5GH upper:
5'-CCAGACCAACAAACACACCA-3'
SP5GH lower:
5'-CCAGCCAGTCAGAGGGAAGA-3'
SP5 Control upper:
5'-GTAACTTGGGACAAGGTAGT-3'
SP5 Control lower:
5'-CACCCTTGAAAATGTAACCC-3'
AXIN2 WRE upper:
5'-CTGGAGCCGGCTGCGCTTTGATAA-3'

AXIN2 WRE lower:
5'-CGGCCCCGAAATCCATCGCTCTGA-3'
AXIN2 Control upper:
5'-CTGGCTTTGGTGAACTGTTG-3'
AXIN2 Control lower:
5'-AGTTGCTCACAGCCAAGACA-3'
CDX2 upper:
5'-CAATGCAGGACAAGGCGATC-3'
CDX2 lower:
5'-GCCGCCATTTGCTCAGTAGTG-3'
GAPDH upper:
5'-5TCGACAGTCAGCCGCATCT-3'
GAPDH lower:
5'-CTAGCCTCCCGGGTTTCTCT-3'
SAT2 upper:
5'-ATCGAATGGAAATGAAAGGAGTCA-3'
SAT2 lower:
5'-GACCATTGGATGATTGCAGTCA-3'

4’Thiouridine-seq

4'Thiouridine-seq was performed in duplicate with dnTCF1EWT cells for the following conditions: untreated (no doxycycline), 2.5 hours doxycycline, and 9.5 hours doxycycline. 4'Thiouridine-seq samples were processed in concert with ChIP-seq samples (one 15 cm plate per 4'Thiouridine-seq replicate). At the time of formaldehyde crosslinking of ChIP-seq samples, 500 µM 4'Thiouridine was added to the cell culture media and incubated with the cells at 37°C for 30 minutes. After 30 minutes, the cells were resuspended in 4 mL Trizol reagent. Total RNA was purified and 4'Thiouridine-labelled RNA was biotinylated (2 µl biotin-HDP [1 mg/mL dissolved in dimethylformamide]/1 µg RNA was added to 1 µl 10x Biotinylation buffer [100 mM Tris pH 7.4, 10 mM EDTA] and 7 µl water) for 1.5 hours at room temperature with rotation. RNA was precipitated and biotinylated RNA was separated using streptavidin-coated magnetic beads (µMacs Streptavidin Kit). Briefly, RNA was heated to 65°C for 10 minutes and immediately placed on ice for 5 minutes. 100 µg of biotinylated RNA was added to 100 µl of streptavidin beads for 15 minutes. µMacs columns were equilibrated with 900 µl Washing buffer (100 mM Tris pH 7.5, 10 mM EDTA, 1M NaCl, 0.1% Tween20) after which the RNA/beads mixture was added. µMacs columns were washed 3 times with 900 µl 65°C Washing buffer and 3 times with 900 µl room temperature washing buffer. 4'Thiouridine-labelled RNA was eluted with 100 mM DTT.

50 ng of 4-Thiouridine-labelled RNA was used to prepare cDNA libraries with the Illumina Tru-Seq kit. Sample preparation began at the elute/frag/prime step and proceeded according to kit specifications. PolyA selection was omitted. Each sample was ligated to a unique linker sequences and multiplexed on the Illumina HiSeq 2500 for a single read 50 bp sequencing run. The 50 bp long reads were trimmed 5 base pairs at the 5' end and then mapped to the human genome (GRCh37/hg19). Reads were mapped to exon-exon junctions using TopHat 2.0.6 (3) with default parameters. TopHat alignments were altered to remove reads that could not be uniquely mapped to the genome. Read counts per gene were extracted using Samtools 0.1.18 (4). Read counts were normalized to the length of the gene and the total number of mapped reads to obtain the normalized transcript abundance (RPKM, reads per kilobase of exon model per million mapped reads). For each time point, two biological replicates were included. The R package edgeR (5) was used to find genes that were differentially expressed between the untreated, 2.5 hours doxycycline, and 9.5 hours doxycycline conditions. Genes with $p<.02$ for the 2.5 hours and $p<.012$ for the 9.5 hours condition were considered to be significantly differentially regulated.

Sequences of Cloned Inserts for Luciferase Assay

GADD45B Insert:

atcttatcatgtctgccatgagccgagcaaaccgtcctcccatggctgcttcctgtaaaccggctcctaaggccagcgggcg
tttgctctcctgacggcacaggaagtcaggctcagcgaattccagctcagagcgggctctggcggcttccaggcagctcca
caatgaggcggctgcgcttttccagtctgctcatcaaagcagagccagccttgtgtggcgcggcgtgccggggtgagtcag
ccccgggggaaggtggccttgaaggcgggagggcggccggcctgcagcagcaacgtgagaacagaagcaaccttc
atctggctctaaaacttccagaaccagtagctttatagctcgcagagctcctggactctttcctcctgggggtctggcaatcgat
ccggggtccgag

TGIF Insert:

Cgtctccccagtaaccgcccggttccagacgaggctcctgccactcaggccgcggagtcgcagcgtcgcccagccccc
ggctcctcctccctgcgtctctcctccctccgctcccggctccccagcggataggagccaccaatcccagccacagacaac
ccttcaaacgaaatagcgaggcggccccctctaacgggcggcgggggcgctggccccctccctgcgccacatctgtccc
gcaccgggcgcagcagctgattcatctgcagccaggtccgggaaggaactgtgctcccggcagttgcagggcagcgtcc
gggcgggtggcttgtccccaggaccgcgctccccccgagccgttttaggtgtgtgttctcttctgtcccgagcagtcagcaaa
ccacagaagaaaaaaatcgtaaggttgctgccttcttagagagacgcttttttccctgttgattcattttagtcctgtaattggaa
aaaccacccaggctttttaaactctggcctccacgcattctaggggc

AXIN2 Insert:

Atggggagtcgtgcggcgggggagggggtgcgggaaacaacaatcccgggttcagagcgaaagccaccggccggg
gagggggaacggcgcgccccgccgccccttttatgcaaaagatccgagcgggcctccgcccccgcccggcccggctttc
aacgcaggtcctgtttccagcagtcactcggcgcaccaaatgtcctccgggcgcttccaacaaaaactgcgctgtggattta
actgtgcacttcaaaggcgcgagccgagcgcacggtcctgaaagggaggtacgcgccggccgcgccccgggccgccc
cccgcagccccgccaccgctccgcagggccgccccgcccgcgcccccctccaggcccggcctccgcct

HIST2H4B Insert:

tgaaggtgttcctggagaatgtgattcgggacgcagtcacctacaccgagcacgccaagcgcaagaccgtcacagccat
ggatgtggtgtacgcgctcaagcgccaggggcgcaccctgtacggcttcggaggctaggccgccgctccagctttgcacg
tttcgatcccaaaggccctttttagggccgaccacttgctcatctgaggagttggacacttgactgcgtaaagtgcaacagta
acgatgttggaaggtaactttggcagtggggcgacaatcggatctgaagttaacggaaagctaccgcggcccatagcgct
cacagccgtaaagacttaagtcgttgaccgaaagcggctttttcacttacctgggctttttttttttttttttttttttttttaaagcctttat
cggtatgaaag

1. Langmead, B. and Salzberg, S.L. (2012) Fast gapped-read alignment with Bowtie 2. *Nat. Methods*, **9**, 357–359.

2. Zhang, Y., Liu, T., Meyer, C.A., Eeckhoute, J., Johnson, D.S., Bernstein, B.E., Nusbaum, C., Myers, R.M., Brown, M., Li, W., et al. (2008) Model-based analysis of ChIP-Seq (MACS). *Genome Biol.*, **9**, R137.

3. Kim, D., Pertea, G., Trapnell, C., Pimentel, H., Kelley, R. and Salzberg, S.L. (2013) TopHat2: accurate alignment of transcriptomes in the presence of insertions, deletions and gene fusions. *Genome Biol.*, **14**, R36.

4. Li, H., Handsaker, B., Wysoker, A., Fennell, T., Ruan, J., Homer, N., Marth, G., Abecasis, G. and Durbin, R. (2009) The Sequence Alignment/Map format and SAMtools. *Bioinformatics*, **25**, 2078–2079.

5. Robinson, M.D., McCarthy, D.J. and Smyth, G.K. (2010) edgeR: a Bioconductor package for differential expression analysis of digital gene expression data. *Bioinformatics*, **26**, 139–140.

Supplementary Figure 1

A) Boxplot of the natural log of peak scores from the top 1000 ChIP-seq peaks from each sample. All differences between samples are significant (t-test p<2.2E-16). B) Overlap (at least one bp) of peaks called in each sample. C) Normalized motif incidence of the WRE (5'-CTTTGWWS-3') and the Helper (5'-RCCGCC-3') in the top 1000 peaks from each ChIP-seq sample (there were only 708 peaks for dnTCF1E^{mut} 0 hours and 354 peaks for dnTCF1E^{mut} 9 hours). D) Irreducibility Discovery Rate (IDR) (31) analysis was performed according to the most recent ENCODE standards (30) (https://sites.google.com/site/anshulkundaje/projects/idr). IDR analysis was performed with MACS2 on biological replicates after 2 hours of dnTCF1EWT and dnTCF1Emut induction. The IDR (y-axis) is equivalent to a stringent FDR for peaks that were reproducibly called in both biological replicates (x-axis). E) Scatter plots for 2 hours of dnTCF1EWT and dnTCF1Emut showing the number of reads for each of the top 1,000 ChIP-seq peaks in one biological replicate versus the number of reads for the same 1,000 peaks in a second biological replicate.

Supplementary Figure 2

A) Histograms of the location of the WRE relative to the peak center in each ChIP-seq sample. The well validated TCF binding element (5'-CTTTGWWS-3') is found near the center of the ChIP-seq peaks. B) The strong-TCF binding element (5'-CCTTTGATST-3') is found near the center of the ChIP-seq peaks.

Supplementary Figure 3

A) Representative ChIP-qPCR on H3K9me3 before and after induction of dnTCF1E^{WT}. The *SAT2* heterochromatic repeat locus serves as a positive control for H3K9me3 and the *GAPDH* promoter serves as a negative control. Induction of dnTCF1E^{WT} did not cause an increase in H3K9me3 at peaks associated with the Wnt target genes *CDX2*, *SP5*, and *AXIN2*. B) Representative ChIP-qPCR on H3K9acetylation. The *GAPDH* promoter serves as a positive control and the *SAT2* heterochromatic repeat region locus serves as a negative control. Induction of dnTCF1E^{WT} did not cause a dramatic decrease in H3K9acetylation at *CDX2*, *SP5*, and *AXIN2* after 2 hours of induction. After 24 hours there was an increase in acetylation at Wnt target genes and surprisingly, also at the *GAPDH* promoter. C) dnTCF1E^{WT} ChIP-qPCR duplicates on WREs associated with the Wnt target genes *SP5 and AXIN2* and control regions that lack WREs. D)

dnTCF1E^{mut} ChIP-qPCR duplicates on the same Wnt responsive and control regions as part C. E, F) ChIP-qPCR validation of peaks that are occupied by both dnTCF1EWT and dnTCF1Emut (*CXADR*, *DACT1*) as well as a C-clamp specific peak occupied only by dnTCF1EWT (*GADD45B*). ChIP extracts were independently prepared as new biological replicates relative to the original ChIP-seq experiment. The ChIP-seq peak assigned to *GADD45B* is confirmed to be C-clamp specific.

Supplementary Figure 4

RSAT *de novo* motif discovery on the top 1000 ChIP-seq peaks from each sample (with the exception of dnTCF1E^{mut} 9 hours, which only had 354 peaks). RSAT has three algorithms that are useful for *de novo* motif finding. The top 3 motifs returned for each algorithm are displayed. The WRE (5'-CTTTGAT-3') was found by at least one algorithm for each sample. The Helper site was found as the top motif in the Positions algorithm for dnTCF1E^{WT} 9 hours and it was the 5th motif returned in the Local Words algorithm for dnTCF1E^{WT} 2 hours (not shown). The Helper site was not found in any dnTCF1E^{mut} *de novo* motifs.

Supplementary Figure 5

A) HOMER *de novo* motif discovery on the indicated samples. B) Differential HOMER *de novo* motif discovery where dnTCF1E^{mut} peaks were used as a background to find motifs comparatively enriched in dnTCF1E^{WT} peaks. C) HOMER optimized PWM for the canonical CTTTGWWS motif using the dnTCF1E^{WT} 2 hours peaks.

Supplementary Figure 6

RSAT differential *de novo* motif discovery. The RSAT Oligo algorithm allows motif enrichment to be determined in one data set versus another dataset. Therefore, the mutant ChIP-seq peaks were used as a background to find motifs that were comparatively enriched in dnTCF1E^{WT} ChIP-seq peaks. All motifs returned from this analysis are displayed. All of the motifs are GC-rich and all of them except one contain embedded Helper sites.

Supplementary Figure 7

A) ChIP-seq peaks with one copy of the WRE and two or more copies of the Helper site (5'-RCCGCC-3') were centered on the WRE. The location of the Helper site relative to

the WRE and the PhyloP 46-way mammalian conservation values (obtained from UCSC) are displayed in a histogram. Helper sites tend to be found within 200bp of the WRE. B) Peak scores associated with regions bound by dnTCF1E^{mut} at both 2 and 9 hours post-induction. There was no significant difference (Wilcoxon-Mann-Whitney test) in WRE or Helper abundance in peaks bound more strongly by dnTCF1E^{mut} at 9 hours post-induction versus 2 hours post-induction.

Supplementary Figure 8

A) 4'Thiouridine-RTPCR replicates on dnTCF1E^{WT} samples. Induction of dnTCF1E caused a decrease in transcription of the Wnt target genes *AXIN2*, *SP5*, and *TNFRSF19*. There was a partial recovery in transcription by 24 hours post-indcution. The 0 hour (no doxycycline), 2 hour, and 9 hours replicates were sequenced. B) 4'Thiouridine-RTPCR on dnTCF1E^{mut} samples.

Supplementary Figure 9

A) Genes that were significantly (2 hours: $p<.012$, 9 hours: $p<.02$) downregulated by dnTCF1E^{WT}-induction at both time points are displayed. There was a significantly greater downregulation of genes at 9 hours post-induction relative to 2 hours post-induction (p=1E-8). B) Known Wnt target genes were significantly more down regulated at 9 hours post-induction compared to 2 hours post-induction. C) There are a greater number of ChIP-seq peaks at 9 hours post-induction associated with a subset of the genes that showed stronger downregulation at 9 hours post-induction.

Supplementary Figure 10

Nucleotide sequences of four regions that were analyzed via subcloning and luciferase assay are shown (Luciferase data shown in Figure 6C). Matches to these motifs are highlighted: canonical WRE motifs (5'-CTTTGWWS-3'; green), the shorter PWM version (5'-CTTTGW-3'; yellow), and Helper sites (5'-RCCGCC-3'; blue). A sequence that would not be detected by our bioinformatic search because of a mismatch in the first position of the motif is highlighted in grey (5'-**G**TTTGAAG-3'). The numbers embedded in the sequence refer to mutations (5'-RCCGCC-3' changed to 5'-TATTAC-3') that were introduced as single (1), double (2) and triple (3) mutations

Supplementary Figure 11

High scoring peaks (MACS peak score > 100) from all WT 2hrs and 9hrs were filtered for those containing: 1. No WRE, 2. At least one WRE, 3. At least one RCCGCC, 4. At least two RCCGCC, 5. At least one RCCGCC and at least one WRE, 6. At least two RCCGCC and at least one WRE, and 7. At least two RCCGCC and no WRE. All RefSeq transcripts were obtained from UCSC build hg19 and were searched for peaks within 30kb of the TSS. Transcripts then were matched to the 4'Thiouridine fold change values for 2 hours and 9 hours, respectively. Boxplots were plotted with the fold change values for any transcripts containing peaks within the groups defined previously. To test for significant differences in gene expression changes, Wilcoxin rank-sum tests were performed between the at least two RCCGCC/at least one WRE group and the at least one WRE group, after removing the transcripts from the at least one WRE group that also have at least two RCCGCC. Similarly, Wilcoxin rank-sum tests were performed between transcripts detected in 4'Thiouridine-seq with WRE-containing peaks (within 30kb of the TSS) and transcripts with peaks within 30kb of the TSS that lack a WRE.

Supplementary Figure 12

Three p32 probes were generated from a sequence taken from the *SP5* promoter, which has two matches to the Helper site (blue) and two WREs (green). *SP5* mut has mutations in the two Helper sites (red). *SP5* methyl has methylated CpGs in the Helper site (4 total methyl groups, 2 on each strand). An Electrophoretic Mobility Shift Assay (EMSA) was carried out using Mock (EVR2) or $TCF1E^{WT}$ expressing COS-1 lysates. Mock COS-1 lysates showed a weak, nonspecific shift of the *SP5* probes. $TCF1E^{WT}$ showed a strong shift of the *SP5* WT probe. There were two shifted bands by $TCF1E^{WT}$ because one band contains two TCF1E molecules bound and one band contains one TCF1E molecule bound. We have previously used a supershift assay to confirm that TCF1E is the responsible agent for the observed shift [15]. Mutation of the two Helper sites caused a decrease in binding by $TCF1E^{WT}$. Methylation of the Helper sites caused an even more dramatic decrease in binding by $TCF1E^{WT}$.

Supplementary Figure 1

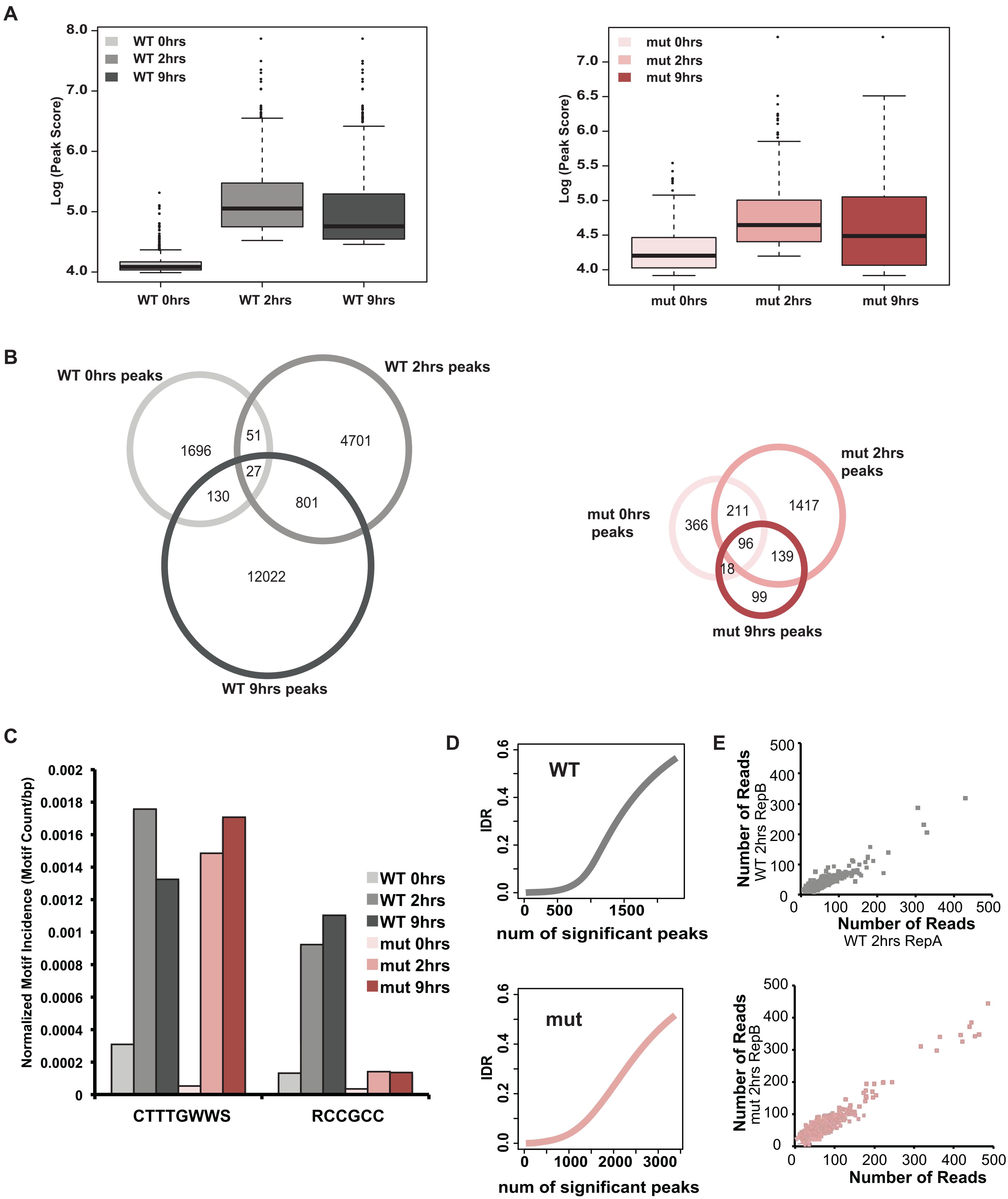

Supplementary Figure 2

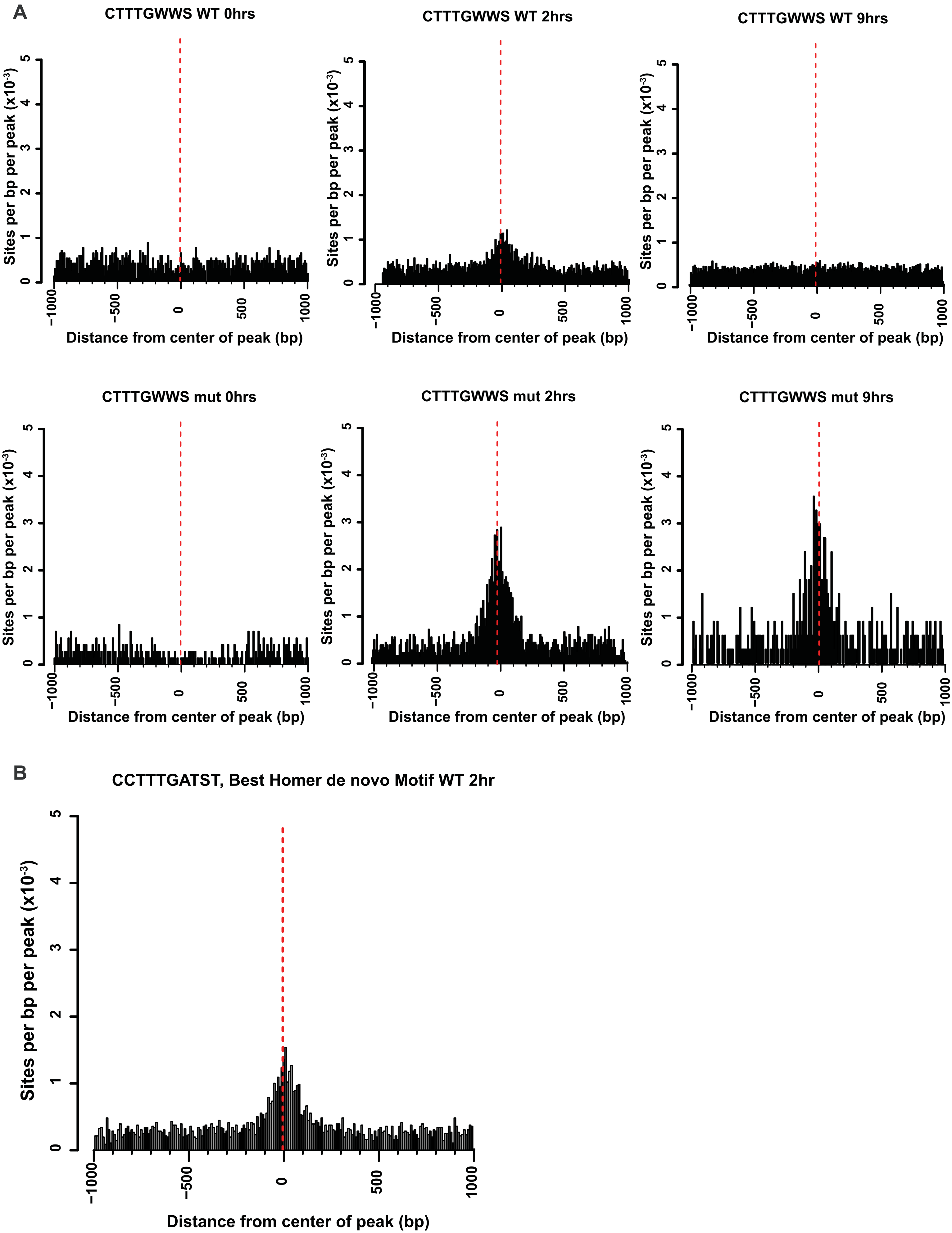

Supplementary Figure 3

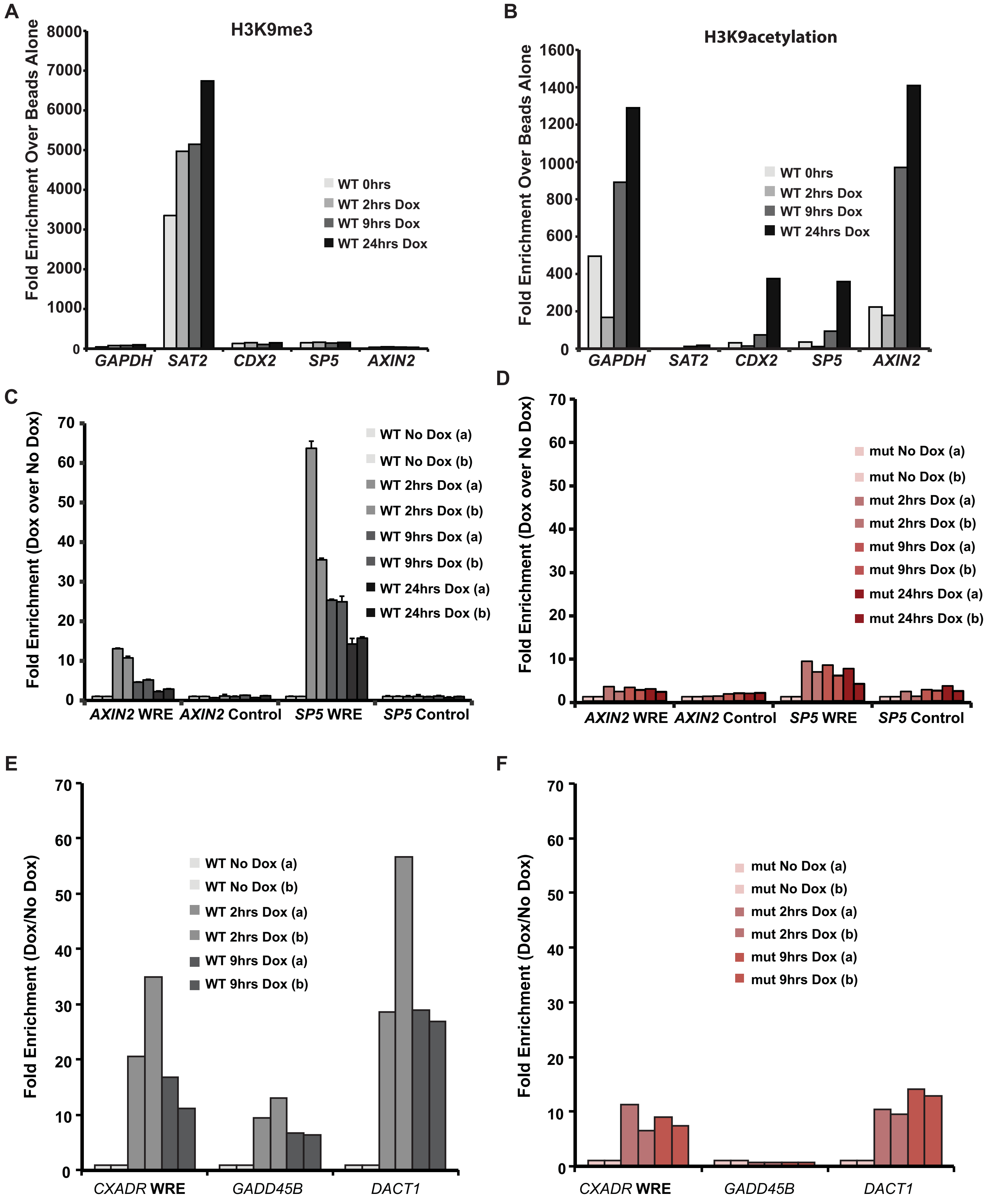

Supplementary Figure 4

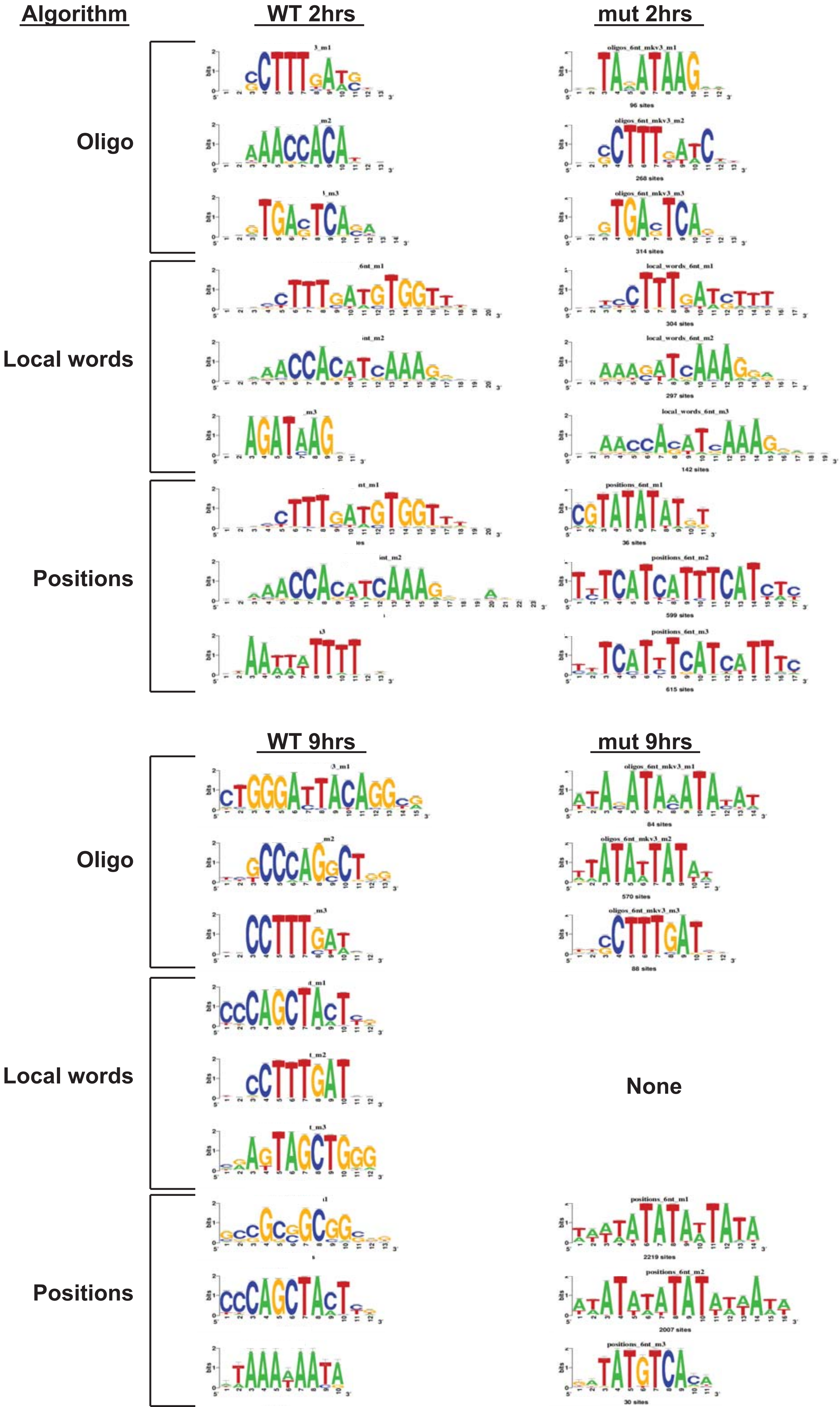

Supplementary Figure 5

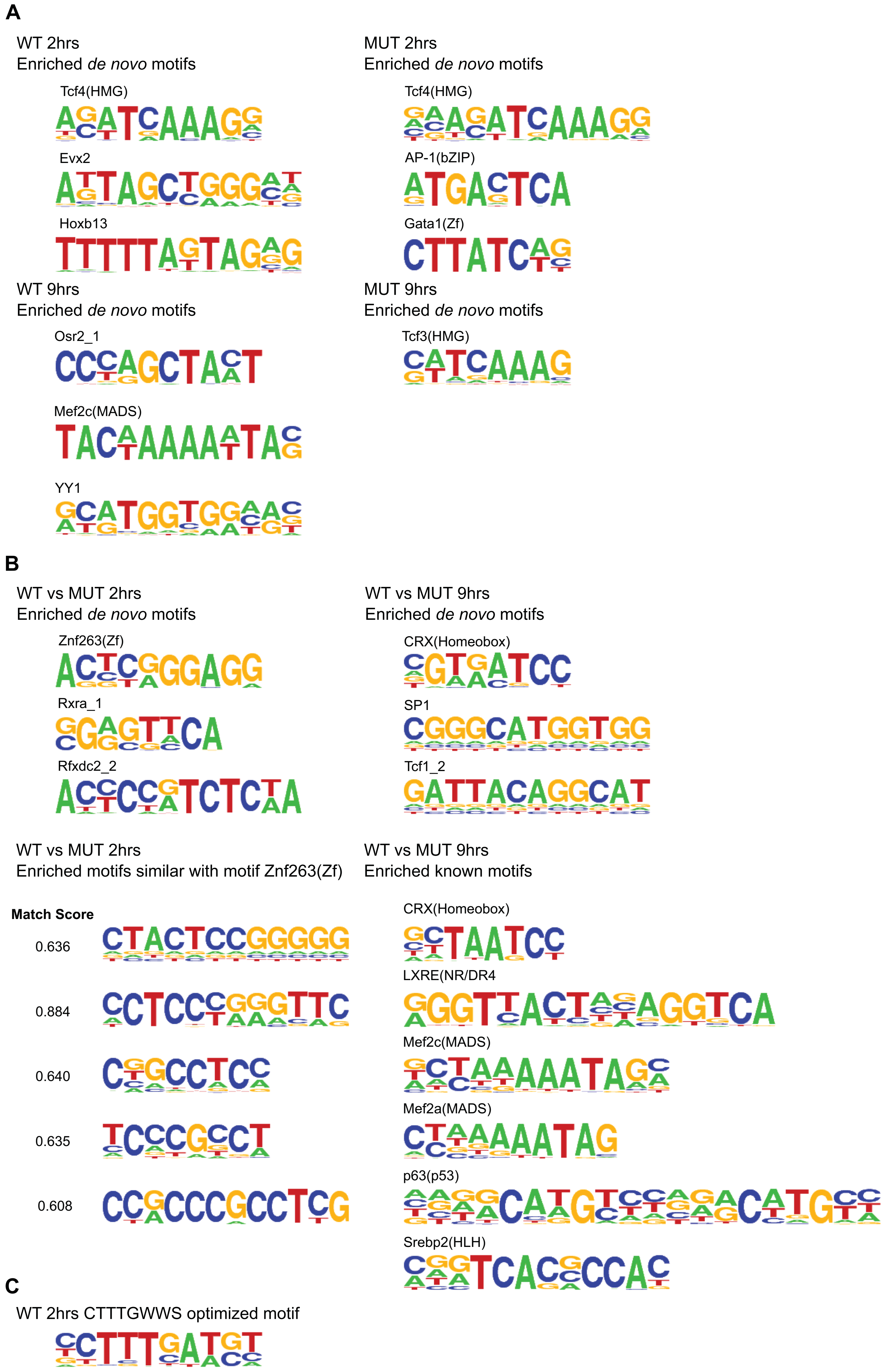

Supplementary Figure 6

WT 2hrs vs. mut 2hrs

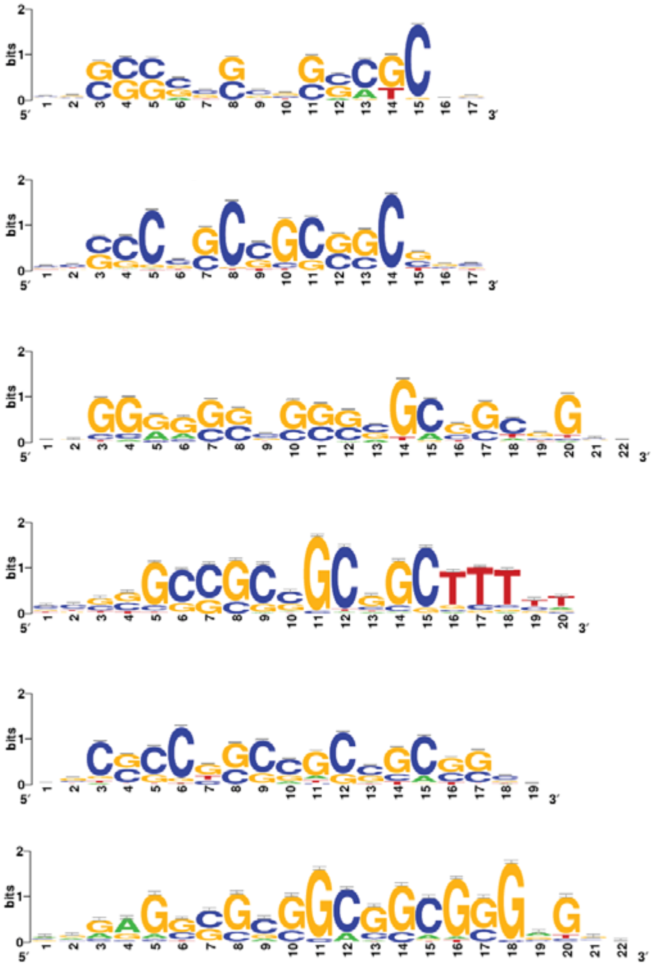

WT 9hrs vs. mut 9hrs

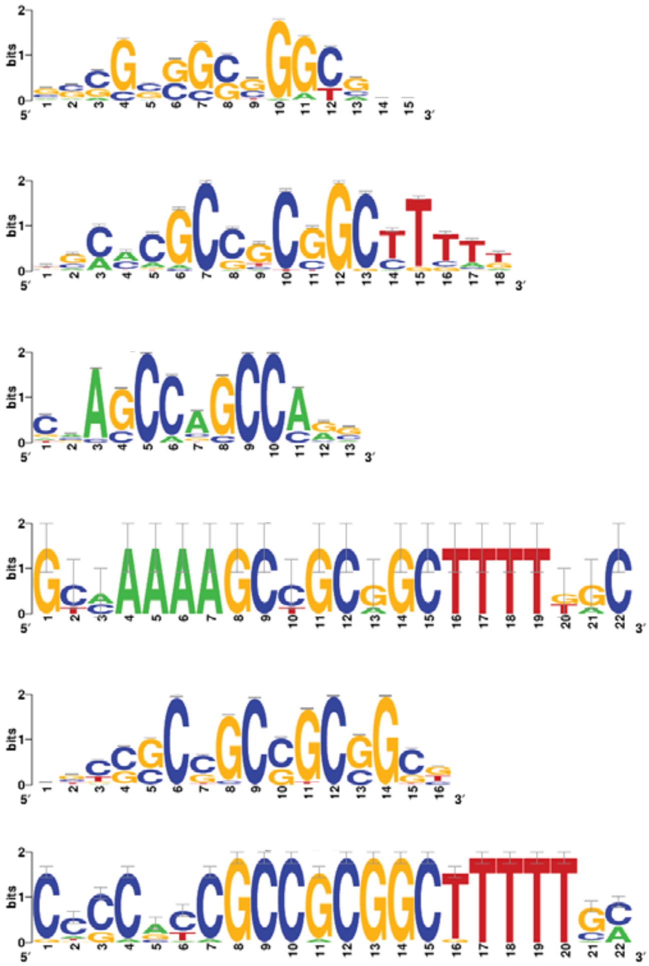

Supplementary Figure 7

A

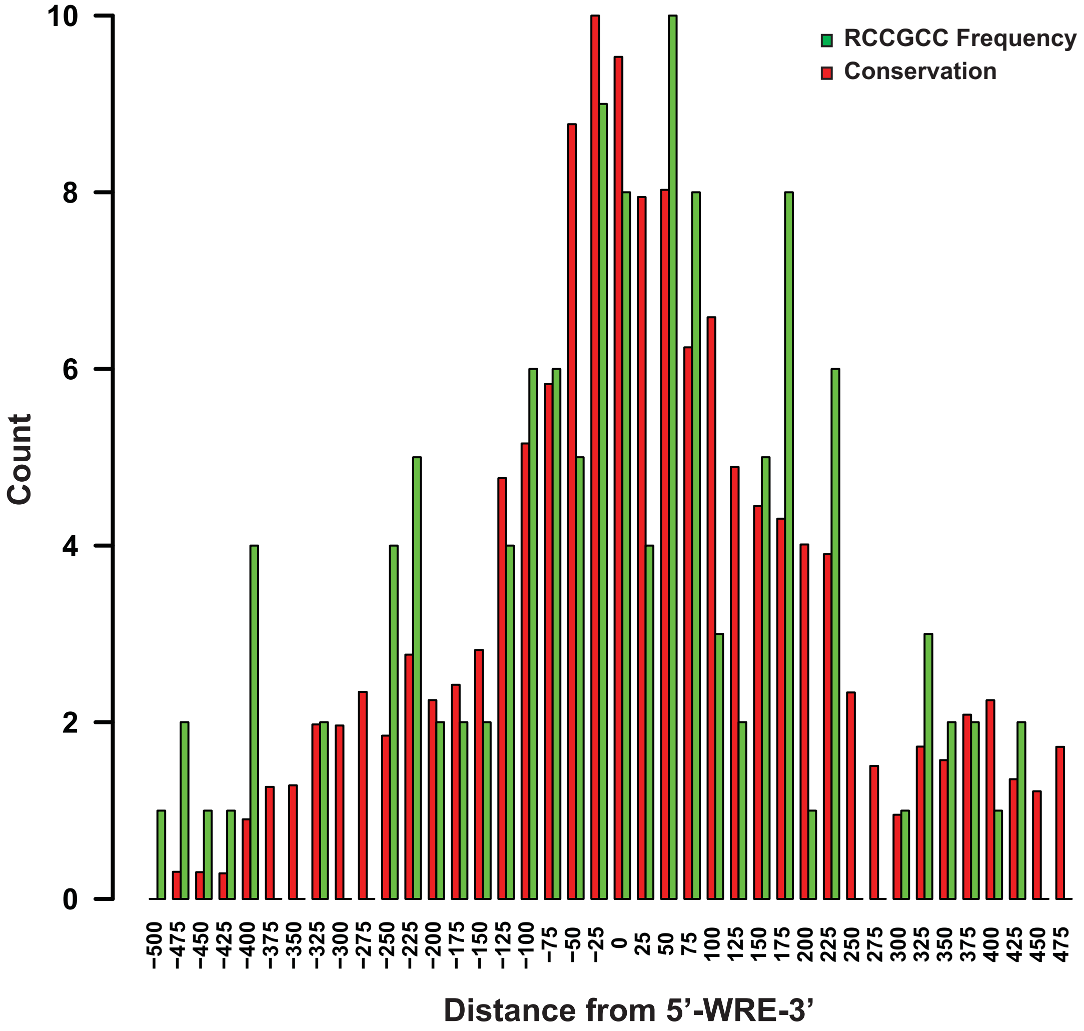

B

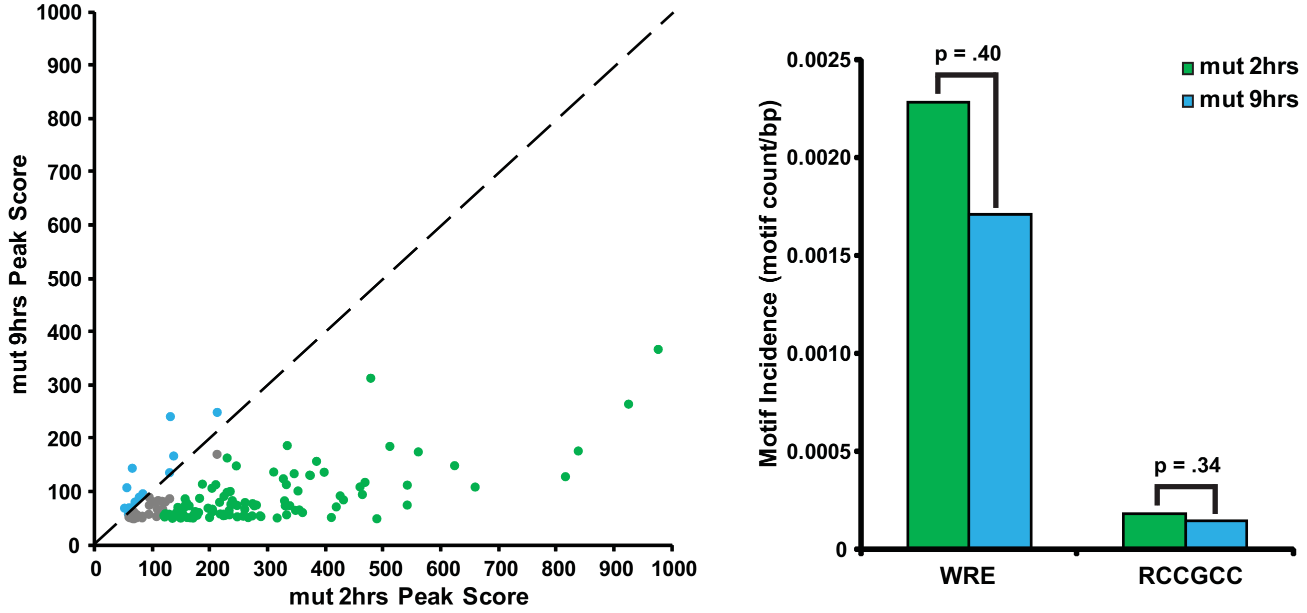

Supplementary Figure 8

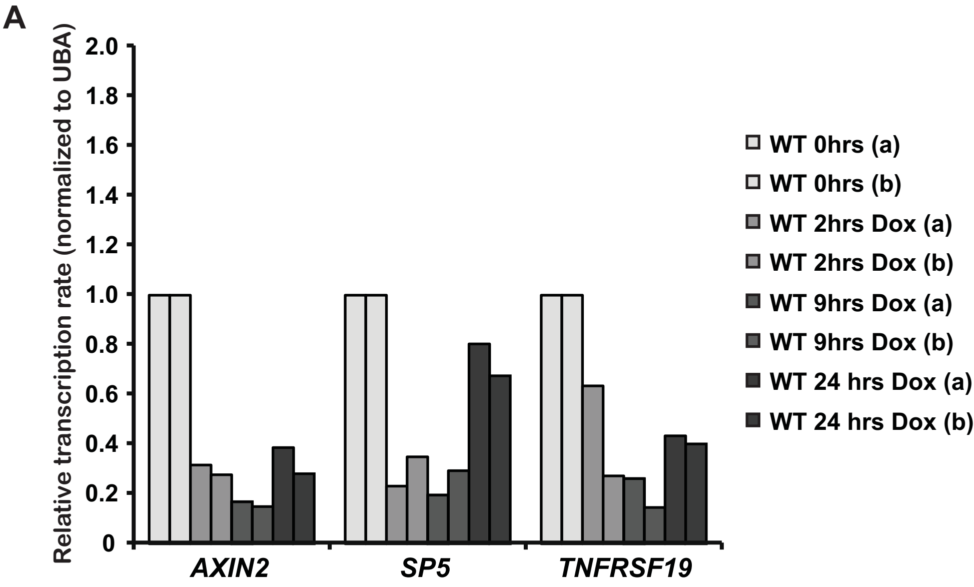

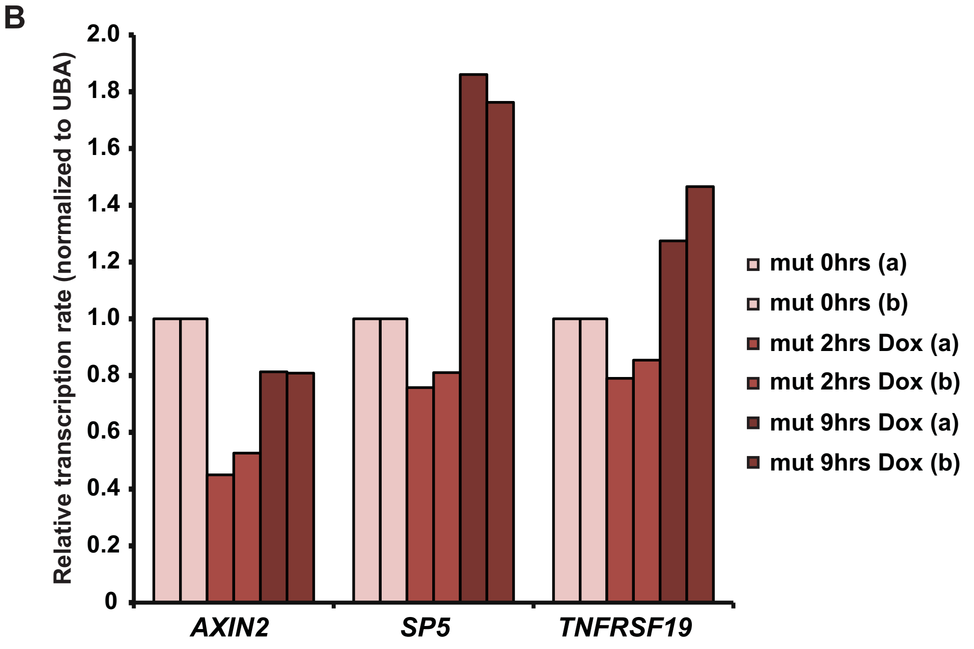

Supplementary Figure 9

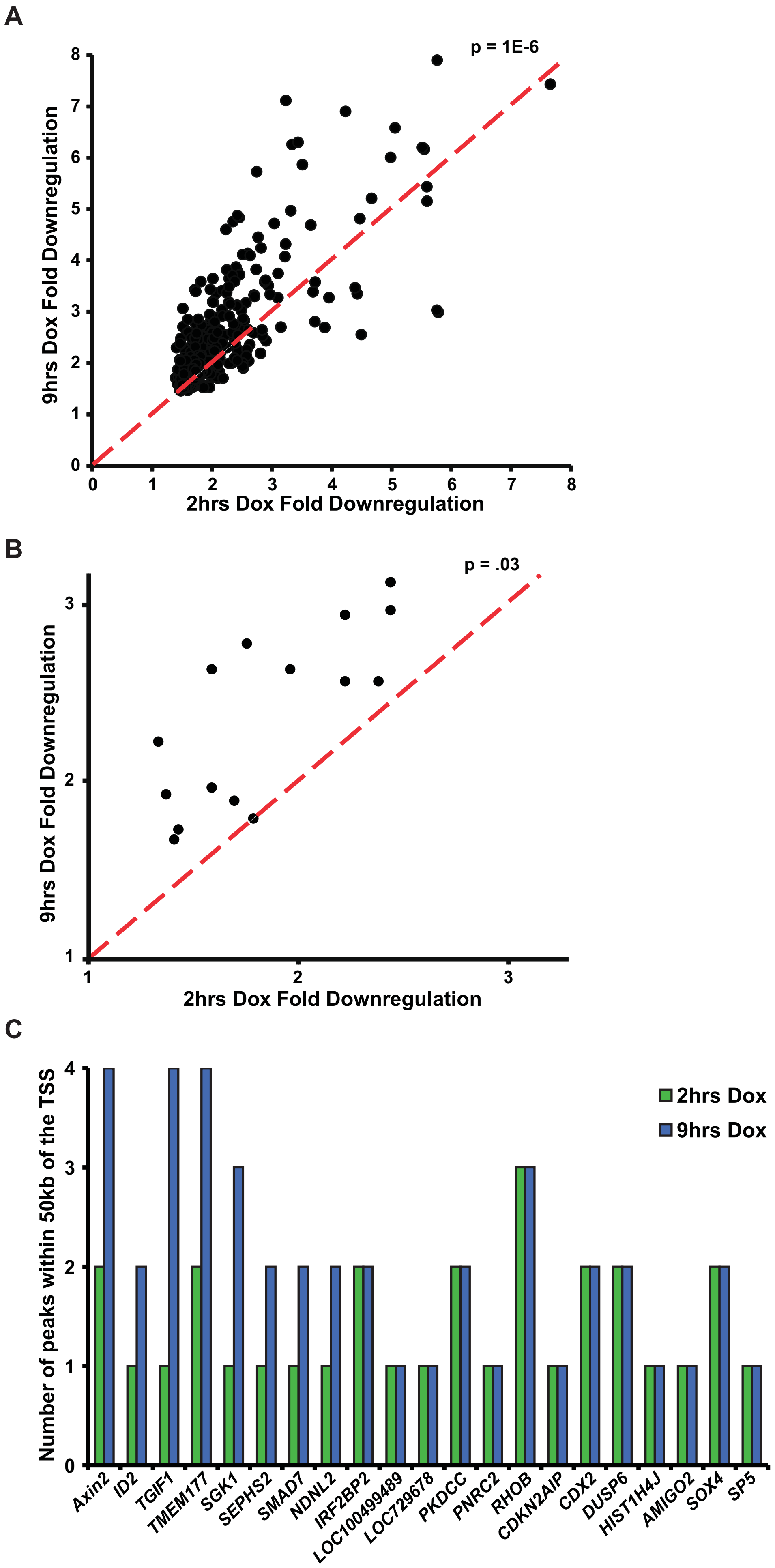

Supplementary Figure 10

RCCGCC **Helper Site**

CTTTGWWS **Wnt Response Element (canonical)**

CTTTGW **Wnt Response Element (short)**

Wnt Response Element (potential)

TopTK: aagatcaaaggggtaagatcaaaggggtaagatcaaaggg

GADD45B: ccatgagccgagcaaaccgtcctcccatggctgcttcctgtaaaccggctcctaaggccagcgggcgtttgctctcctgacggcacag
gaagtcaggctcagcgaattccagctcagagcgggctctggcggcttccaggcagctccacaatgaggcggctgcgcttttccagtctgctcatcaaagc
agagccagccttgtgtggcgcggcgtgccggggtgagtcagccccgggggaaggtggccttgaaggcgggaggggcggccggcctgcagcagca
acgtgagaacagaagcaaccttcatctggctctaaaacttccagaaccagtagctttatagctcgcagagctcctggactctttcctcctgggggtctggca
atcgatccggggtccgag

AXIN2: atggggagtcgtgcggcgggggagggggtgcgggaaacaacaatcccgggttcagagcgaaagccaccggccggggaggggaa
cggcgcgccccgccgcccttttatgcaaaagatccgagcgggcctccgcccccgcccggcccggctttcaacgcaggtcctgtttccagcagtcact
cggcgcaccaaatgtcctccgggcgcttccaacaaaaactgcgctgtggatttaactgtgcacttcaaaggcgcgagccgagcgcacggtcctgaaa
gggaggtacgcgccggccgcgccccgggccgccccccgcagccccgccaccgctccgcagggccgccccgcccgcgccccctccaggcccg
gcctccgcct

HIST2H4B: tgaaggtgttcctggagaatgtgattcgggacgcagtcacctacaccgagcacgccaagcgcaagaccgtcacagccatggatgtg
gtgtacgcgctcaagcgccaggggcgcaccctgtacggcttcggaggctaggccgccgctccagctttgcacgtttcgatcccaaaggcccttttaggg
ccgaccacttgctcatctgaggagttggacacttgactgcgtaaagtgcaacagtaacgatgttggaaggtaactttggcagtggggcgacaatcggat
ctgaagttaacggaaagctaccgcggcccatagcgctcacagccgtaaagacttaagtcgttgaccgaaagcggctttttcacttacctgggctttttttttt
ttttttttttttttttaaagcctttatcggtatgaaag

TGIF: cgtctccccagtaaccgcc(3)cggttccagacgaggctcctgccactcaggccgcggagtcgcagcgtcgcccagcccccggctcctcctcc
ctgcgtctctcctccctccgctcccggctccccagcggataggagccaccaatcccagccacagacaacccttcaaacgaaatagcgaggcggc(1)c
ccctctaacgggcggc(2)gggggcgctggccccctccctgcgccacatctgtcccgcaccgggcgcagcagctgattcatctgcagccaggtccggg
aaggaactgtgctcccggcagttgcagggcagcgtccgggcgggtggcttgtccccaggaccgcgctccccccgagccgttttaggtgtgtgttctcttctg
tcccgagcagtcagcaaaccacagaagaaaaaaatcgtaaggttgctgccttcttagagagacgcttttttttccctgttgattcattttagtcctgtaattggaa
aaaccacccaggctttttaaactctggcctccacgcattctaggggc

Supplementary Figure 11

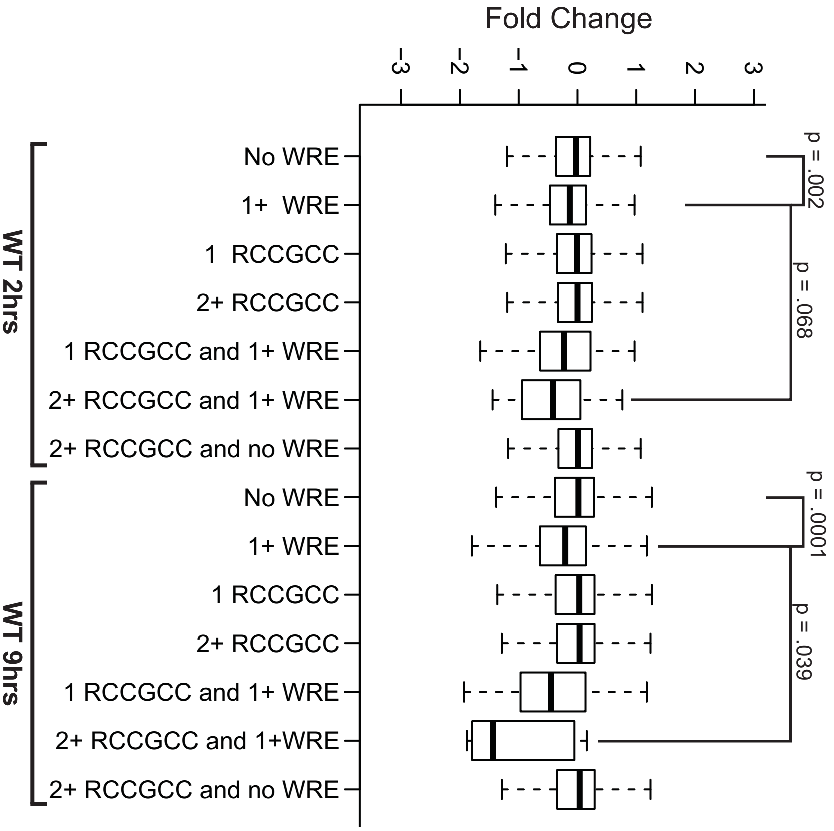

Supplementary Figure 12

SP5 WT AGCCGCTATTCTTTGATGATTGGGTAGCGGCAAACTTCAAAGCC

SP5 mut AGTATATATTCTTTGATGATTGGGTAGTTATAAACTTCAAAGCC

SP5 methyl AGCC*GCTATTCTTTGATGATTGGGTAGC*GGCAAACTTCAAAGCC

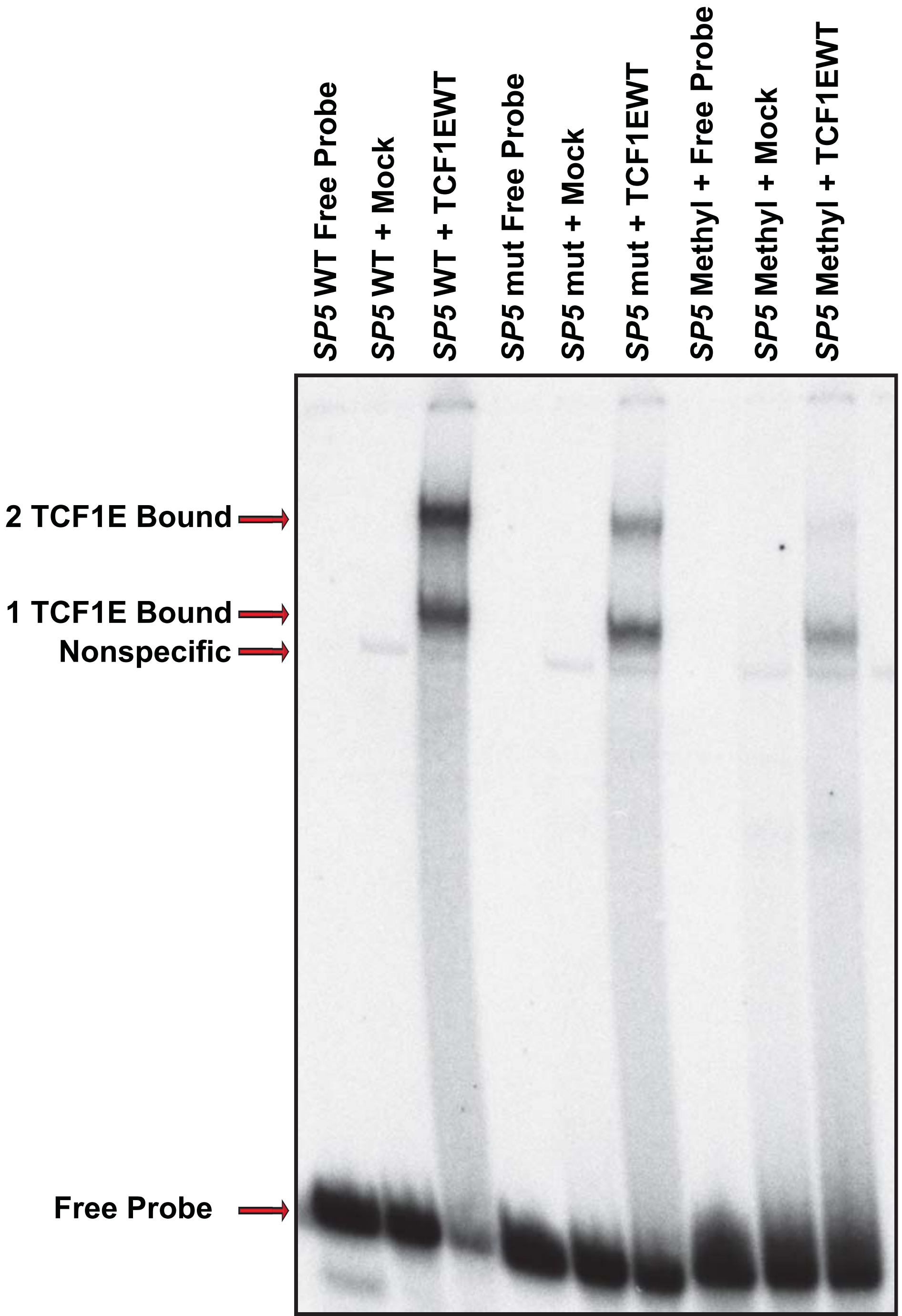

Supplement: SUPPLEMENTARY DATA [file supp_gku1186_nar-00699-x-2014-File016.pdf]
